# Hematological Parameters and Mercury Exposure in Children Living Along Gold-Mining-Impacted Rivers in the Mojana Region, Colombia

**DOI:** 10.1007/s12011-025-04557-6

**Published:** 2025-03-01

**Authors:** Jenny Palomares-Bolaños, Karina Caballero-Gallardo, Jesus Olivero-Verbel

**Affiliations:** 1 Functional Toxicology Group, School of Pharmaceutical Sciences, Zaragocilla Campus, University of Cartagena, 130014 Cartagena, Colombia; 2 Environmental and Computational Chemistry Group, School of Pharmaceutical Sciences, Zaragocilla Campus, University of Cartagena, 130014 Cartagena, Colombia

**Keywords:** Heavy metal, Health, Immunotoxicity, Inflammation, Contamination

## Abstract

Mercury (Hg) exposure is a key determinant of human health, arising from environmental, occupational, and domestic sources, as well as the consumption of contaminated food, particularly fish. Due to their developmental stage and heightened susceptibility, children are especially vulnerable. This study evaluated Hg contamination in a high-risk pediatric population affected by local economic activities such as small-scale gold mining, agriculture, and fishing. The objective was to determine whether exposure to this toxic metal was associated with alterations in hematological biomarkers, analyzing potential correlations with total Hg (T-Hg) levels in the hair and blood of 282 children aged 6 to 12 years in the Mojana Bolivarense (Colombia). The mean T-Hg concentration in hair was 0.88 µg/g in Magangue, 1.55 µg/g in Achi, and 0.26 µg/g in Arjona. A total of 35.0% and 44.4% of the examined minors from Magangue and Achi, respectively, exceeded the international threshold for hair Hg (1 µg/g). Likewise, blood T-Hg concentrations surpassed the recommended safety level (5 μg/L) in 39.4% of children from Achi and 0.8% from Magangue, while in Arjona, all values remained below this threshold. The geometric mean (GM) values of the hair-to-blood Hg ratio varied across locations. Spearman correlations revealed a very strong positive association between blood and hair T-Hg levels in Achi (*ρ* = 0.801; *p* < 0.01), a moderate correlation in Magangue (*ρ* = 0.325; *p* < 0.01), and some significant links with hematological parameters. Multiple linear regression analysis indicated a positive association between blood T-Hg concentration and white blood cell count (*β* = 0.053; *p* = 0.021) and granulocytes (*β* = 0.086; *p* = 0.011), as well as an inverse correlation with lymphocyte percentage (*β* =  − 0.353; *p* = 0.036). These findings suggest that Hg exposure may influence inflammatory and immunosuppressive processes, posing a potential health risk to vulnerable populations, particularly young individuals.

**Supplementary Information:**

The online version contains supplementary material available at 10.1007/s12011-025-04557-6.

## Introduction

Mercury (Hg) pollution is a pressing environmental issue, particularly in regions where gold mining activities are prevalent [1, 2]. These activities often involve the use of Hg in the extraction process, leading to the release of elemental Hg and its subsequent conversion to methylmercury (MeHg), a highly toxic form that readily accumulates in aquatic ecosystems [3]. The contamination of rivers and water bodies in gold mining-affected areas has raised concerns about the potential health risks for both humans [4] and wildlife [5]. Methylmercury, once ingested, can enter the food chain and subsequently impact local communities, especially those residing near riversides [2, 3]. Consequently, within mining regions, the presence of this element can be identified in in water [6], air [4], superficial soil [7], and sediments [4]. Furthermore, elevated concentrations of total Hg (T-Hg), exceeding the permissible levels established by the World Health Organization [8], have been detected [4].

Presently, substantial evidence strongly links Hg contamination in the environment to gold mining processes. This conclusion is drawn from biomonitoring studies conducted across numerous countries, including Ghana [9], Indonesia [10, 11], Brazil [12], Peru [13], Ecuador [14], and Colombia [1, 4, 6, 7], among other locations. In addition, ecotoxicological studies reveal the biomagnification of MeHg throughout biological species within an ecosystem’s food chain. With an inherent toxicokinetic potential characterized by easy absorption, distribution, and cell uptake, MeHg adversely affects organisms [15–17]. Likewise, the correlations between concentrations of Hg in humans and the consumption of fish from gold mining areas have gained increasing significance. Diets based on fish contaminated with Hg promotes its accumulation in hair [4, 18, 19], blood [20], and urine [21], often surpassing the maximum level acceptable stipulated of these matrices [22, 23].

Communities living in close proximity to gold mining-contaminated riversides are at an increased risk of mercury exposure due to their reliance on local water sources and consumption of aquatic resources such as fish [12, 19, 24]. Among the exposed populations, children are particularly vulnerable due to their developing physiology and unique behaviors that may lead to higher exposure rates. Moreover, the immaturity of their metabolic and detoxification processes makes them less capable of effectively eliminating Hg from their bodies and more prone to adverse health effects [25, 26]. As a result, understanding the potential health effects of Hg exposure on children becomes paramount, necessitating a comprehensive investigation into various health impact domains.

The exposome of children remains vulnerable to environmental contamination, especially those residing in territories marked by mining activities, where fish stands as a primary food source. Adverse effects stemming from such exposure can manifest in children over the short, medium, or long term, contingent upon the extent of exposure and their developmental stage. Furthermore, there exists the potential for such effects to be transmitted during fetal growth through the umbilical cord and maternal placenta [27, 28]. In this regard, Hg can trigger disturbances within the central nervous system [29], as well as in the lungs, liver, and other vulnerable organs [30].

Measurements of T-Hg concentrations in blood has been extensively used to monitor exposure to Hg. Blood also served as a valuable indicator of physiological responses to environmental stressors, including heavy metal exposure. Mercury’s detrimental effects on hematological parameters have been widely documented, with studies suggesting correlations between mercury exposure and alterations in blood cell-related parameters [31]. The examination of hematological variables provides insights into potential disruptions in oxygen-carrying capacity, immune function, and overall well-being. Consequently, a comprehensive analysis of these parameters in children exposed to Hg contributes to a holistic understanding of the health implications of such pollution. Other authors suggest that exposure to Hg is linked to attention-deficit hyperactivity disorder (ADHD) in children [32], cardiovascular system disorders [33], cognitive disorders, memory, language, motor skills, and behavioral functions [34]. Furthermore, the expression of genes, enzymes, and human polymorphisms demonstrates the susceptibility of children to neurological deficits and cognitive development issues among those who may have been exposed to toxic Hg [35].

Regarding the main health impacts, the possible relationship between the presence of heavy metals in the body and alterations in red blood cells, as well as various abnormalities in the white blood cell count, has been evaluated in populations potentially exposed to methylmercury through fish consumption [31]; additionally, the possibility that the mechanisms of action of Hg affect red blood cells, white blood cells, and immune system cells has been explored [36]. Consequently, parameters such as hemoglobin concentration (HGB), hematocrit (HTC), red blood cell (RBC), white blood cell (WBC), mean corpuscular volume (MCV), mean corpuscular hemoglobin (MCH), and mean corpuscular hemoglobin concentration (MCHC) are examined to assess the toxic stress induced by metals in biological models [37]. Although the hematotoxicity of Hg in humans has not been fully established, it has been studied that, indirectly, the presence of traces of mercury in the body can cause serious conditions such as anemia, since abnormalities in red blood cells are associated with problems of hemolysis [38–40].

Similarly, research suggests that the presence of Hg and its organic forms affects lymphocytes and monocyte concentrations, causing immunostimulation and lymphoproliferation due to the sensitivity of the hematological system to toxic agents [41]. However, scientific evidence in this field remains limited, making observational studies useful and necessary to identify associations between variables related to Hg exposure, its impacts, and the prevalence of these hypotheses, particularly in vulnerable pediatric populations. This study aimed to evaluate Hg exposure in a vulnerable pediatric population affected by environmental contamination due to local economic activities such as small-scale gold mining, agriculture, and fishing. The objective was to determine whether exposure to the toxic metal was associated with alterations in hematological markers as determinants of health by examining possible associations with total Hg concentrations found in the hair and blood of children living in the Mojana region (Colombia).

## Materials and Methods

### Study Area

**Fig. 1:**
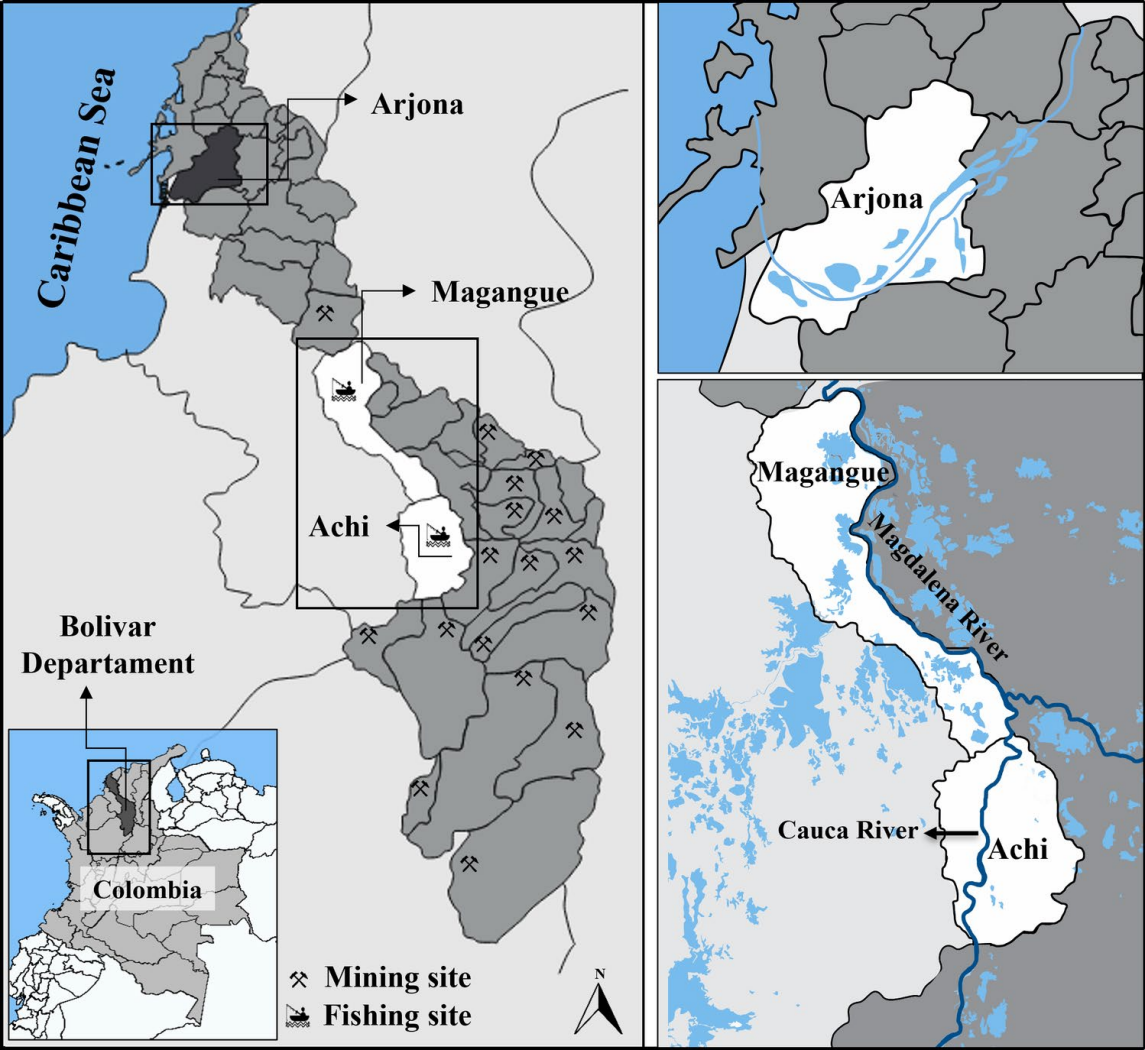
Geographical location of the study area

In Colombia, the Bolivar department comprises 2.3% of the country’s total territory. This department is composed of 45 municipalities and 2 districts, organized into 6 economic and social development zones. The present study was conducted in the Mojana Bolivarense, a subregion that accounts for 13.8% of the Bolivar department’s land area. This covers a total area of 6143 square kilometers and includes the municipalities of Achi, Magangue, Montecristo, Pinillos, San Jacinto, and Tiquisio. This area is known for its rich biodiversity [42] and important wetland ecosystems, but it has also faced challenges related to mercury exposure due to gold mining in the region [2]. This particular zone of economic and social development primarily relies on agriculture and aquaculture activities, thanks to its access to important water sources in the nation, the Magdalena and Cauca Rivers. The selected sampling points are located in the municipalities of Magangue (9°14′48″ N, 74°45′34″ W) and Achi (8°34′09″ N, 74°33′22″ W), both urban areas situated along the banks of the Magdalena and Cauca rivers, respectively (Fig. 1). Magangue is one of the most populous cities in the department, where agriculture contributes significantly to the country’s gross domestic product through the cultivation of crops such as corn, yams, and rice. Conversely, the municipality of Achi is renowned for its abundant water resources, supporting agricultural production and fishing. Additionally, for comparative purposes, biological samples were also collected from the municipality of Arjona (10°15′18″ N, 75°20′41″ W), a town whose urban area has lower influence from the Magdalena River.

### Selection of Participants and Data Collection

The study participants consisted of children aged between 6 and 12 years from three municipalities in the Bolivar department of Colombia: Magangue, Achi, and Arjona. A total of 282 children were recruited for the study between January 2021 and February 2022. Participants were selected based on the criterion that they were the offspring of fishermen, farmers, and miners residing in the area. The recruitment process involved community outreach and engagement, with the cooperation of municipal leaders. In all cases, both parents and children provided written consent and assent forms, respectively, following the guidelines approved by the Ethics Committee of the University of Cartagena (CE-119, 2019). Following participant selection, a survey was administered to gather sociodemographic information, and anthropometric data were recorded.

### Collection of Biological Samples

Approximately 50–100 mg of hair from the participants was taken from the occipital region of the skull, obtaining a 1-cm-thick portion. The sample obtained was stored in a white paper envelope duly labeled with code, name, ID and date, following national and international recommendations for the collection of this type of human matrix [2]. Once in the laboratory, the samples were cut with sterilized scissors in order to homogenize, stored in a 15 mL falcon where they were subjected to washing with 2% Triton X-100, deionized water, acetone and centrifuged to eliminate interference. The washed hair samples were sealed with aluminum foil, dried in an oven at 40 °C for 12 h, and stored in desiccators until analysis.

In the case of blood samples, these were collected from each participant collection tubes with ethylenediaminetetraacetic acid-EDTA (Vacutainer ®) as anticoagulant (682030202) for subsequent analysis (Analysis of Hg and hematological parameters) [43, 44]. Once obtained, they were stored in thermal boxes at an approximate temperature of 4 °C and transported to the laboratory for processing. Hemograms were performed immediately with one of the collected blood tubes, and another was stored at − 80 °C for Hg analysis.

### Mercury Analysis in Blood and Hair

Total Hg concentrations in hair and blood samples were measured using a Direct with a Milestone Direct Mercury Analyzer (DMA-80; Milestone, Sorisole, Italy), by the combustion, amalgamation, and atomic absorption spectrometry technique, using the U.S. Environmental Protection Agency (U.S. EPA) method 7473 [45]. The quality control for the measurements was carried out using calibration curves made with the certified material IAEA-085 of the International Atomic Energy Agency (Analytical Quality Control Services, Vienna, Austria), with a linearity ≥ 0.99, a relative standard deviation (RSD) lower than 10% and percentage recovery of 88.5–113.9% when using the certified fish muscle sample ERM-BB422 (European Reference Material, Institute for Reference Materials and Measurements, Joint Research Centre). In the case of blood samples, the reference material BE12-12,13,14,15 (Wadsworth Center, Trace elements in whole blood) was used [20]. The detection limits for hair and blood using preheated empty combustion canisters as blanks, were 0.007 μg/g and 0.006 μg/L, respectively.

### Hematological Analysis

An electrical impedance automated blood count was performed to quantify 18 hematological parameters (WBC count; RBC count; HGB; HTC; MCV; MCH; MCHC; total platelet (PLT) count; plateletcrit (PCT); mean platelet volume (MPV); platelet distribution width (PDW); red cell distribution width (RDW); lymphocyte (LYM) count; granulocyte (GRA) count; lymphocyte percentage (LYM%); granulocyte percentage (GRA%)). For this purpose, the Abacus 380 Automated equipment was used, employing a reference blood sample for calibrations and quality control as published elsewhere [44, 46]. Samples were analyzed twice.

### Statistical Analysis

The data for qualitative variables are presented with their frequency distribution. Quantitative variables are described using descriptive statistics and expressed as mean ± standard deviation. The Kolmogorov–Smirnov test was performed to evaluate the normality of the data. Variables that did not show a normal distribution were normalized using natural logarithmic transformation of the values for subsequent multiple regression analysis. Categorical variables (sex, schooling level, canned fish intake, fish consumption) were analyzed using the chi-square test (*X*^2^) to compare observed distributions between sites. The Kruskal–Wallis (KW) test for more than two groups was used to evaluate differences among the three sites for age, weight, height, BMI, and T-Hg concentrations in hair and blood, followed by Dunn’s post hoc test. Additionally, the Mann–Whitney test was used to compare the mean T-Hg concentrations in hair and blood between sexes.

The relationship between T-Hg concentrations in hair, blood, and hematological parameters by sex was evaluated using Spearman’s correlation for the three sampling sites, followed by multivariate linear regression (MLR) analysis to assess the associations of T-Hg with hematological parameters. The regression model was adjusted for confounding variables (age, sex, and sampling site). A QQ plot was used to establish the normality of residuals. Multicollinearity was assessed using the variance inflation factor (VIF), which was < 10. A homoscedasticity plot was also used to determine the homoscedasticity of residuals, suggesting constant variance in all cases. The independence of observations in the MLR was evaluated using a residual vs. order plot, providing evidence that residuals are distributed randomly without any trend or dependence. For all statistical tests, significance was set at *p* < 0.05. Initial data processing was conducted in Microsoft Excel, and statistical tests were performed using GraphPad Prism 8 and IBM SPSS 26 statistical software.

## Results

### General Characteristics of the Participants

Two hundred eighty-two children from Magangue (*n* = 123), Achi (*n* = 99), and Arjona (*n* = 60) voluntarily provided hair and blood samples for their participation in this research. Information regarding various demographic and health-related variables across the three sampling sites is presented in Table 1. The sex distribution remained consistent across these diverse sampling locations, with an overall composition of 55% male participants and 45% female participants. The M/F ratio was 1.22. The mean age of the entire cohort was 9.2 ± 0.2 years. The results indicate significant differences in ages among the three sampling sites. Magangue showed a significantly higher average age compared to Arjona (the reference site), with means of 9.5 ± 0.2 and 9.0 ± 0.2 years, respectively (*p* < 0.05). In contrast, no significant differences were observed in sex distribution, weight, or height among the sampling sites (*p* > 0.05). The mean BMI value (16.5 ± 0.1) indicated underweight (< 18.5), but BMI significantly varied among the sampling sites, with Achi exhibiting the highest value (GM = 16.8). In terms of education, the majority of individuals have completed primary school, with similar distributions across the sampling sites. Canned fish consumption did not vary between sampling sites. Various characteristics related to the parents of the children, such as occupation, education level, alcohol, and tobacco consumption, vary according to the study groups (Supplementary Table S1). Regarding characteristics related to children’s early years, no significant associations were observed among the sampling sites for the type of delivery, breast milk consumption, and premature delivery (Supplementary Table S2). The data also indicate that premature deliveries were more frequent in Achi (10.1%) compared to Arjona (3.3%) and Magangue (3.3%).

**Table 1:** General characteristics of children by sampling site

Variable	Strata	Total, *n* = 282	Sampling sites			Statistic	*p* value
			Arjona, *n* = 60	Magangue, *n* = 123	Achi, *n* = 99		
Age (years)	Mean ± SEM	9.2 ± 0.2	8.7 ± 0.2	9.5 ± 0.2	9.0 ± 0.2	KW = 7.5	0.02*
	GM	8.9	8.5	9.3	8.8		
Sex	*Male*	155 (55.0)	28 (46.7)	64 (52.0)	63 (63.6)	*X*^2^ = 5.5	0.06
	*Female*	127 (45.0)	32 (53.3)	59 (48.0)	36 (36.4)		
Weight (kg)	Mean ± SEM	29.7 ± 0.5	29.8 ± 1.1	28.8 ± 0.7	30.5 ± 0.9	KW = 1.7	0.41
	GM	28.7	28.6	27.9	29.4		
Height (cm)	Mean ± SEM	133.0 ± 0.7	134.1 ± 1.8	132.4 ± 1.0	132.5 ± 1.2	KW = 0.5	0.77
	GM	132.5	133.4	131.9	132.0		
BMI (kg/m^2^)	Mean ± SEM	16.5 ± 0.1	16.3 ± 0.4	16.1 ± 0.2	16.9 ± 0.3	KW = 6.5	0.04*
	GM	16.3	16.1	16.0	16.8		
Schooling level	Preschool	37 (13.1)	13 (21.7)	16 (13.1)	8 (8.1)	*X*^2^ = 12.1	0.06
	Primary school	195 (69.1)	35 (58.3)	92 (75.4)	68 (68.7)		
	Secondary school	49 (17.8)	12 (20.0)	14 (11.5)	23 (23.2)		
Canned fish intake	Yes	171 (60.6)	32 (53.3)	74 (60.2)	65 (65.7)	*X*^2^ = 2.4	0.30
	No	111 (39.4)	28 (46.7)	49 (39.8)	34 (34.3)		
Fish consumption *(meals/week)*	1–4	111 (76.5)	46 (100.0)	27 (49.1)	38 (86.4)	*X*^2^ = 39.6	< 0.01*
	5–10	21 (14.5)	0 (0.0)	17 (30.9)	4 (9.1)		
	> 10	13 (9.0)	0 (0.0)	11 (20.0)	2 (4.5)		

The data is presented as the mean ± standard error of the mean or as frequencies *n* (%), depending on the variable.

GM, geometric mean; KW, Kruskal–Wallis test followed by Dunn’s multiple comparison test; *X*^2^, chi-square test.

*Statistical significance (*p* < 0.05)

.

### T-Hg Concentrations in Hair and Blood

**Fig. 2:**
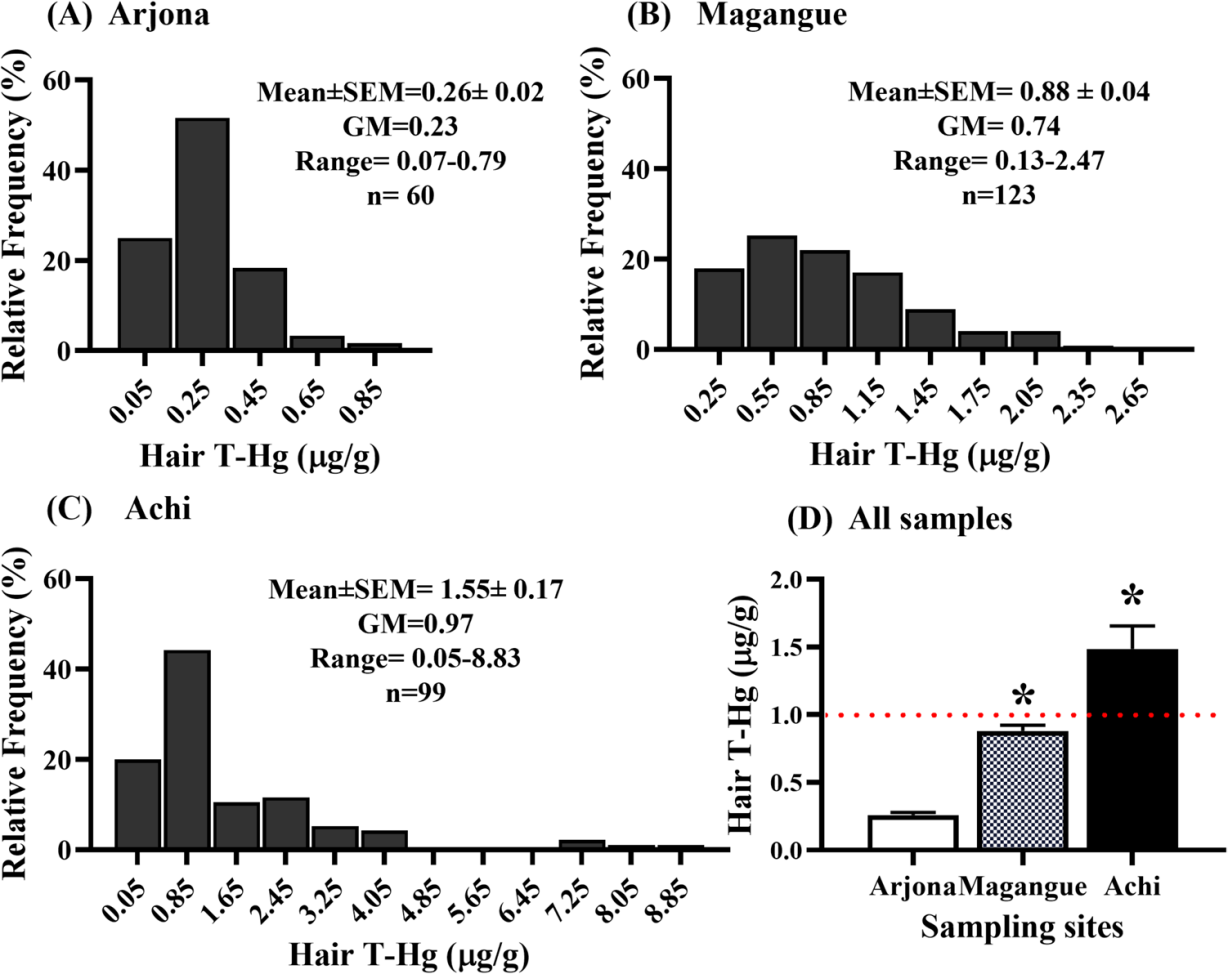
Frequency distribution (**A**–**C**) and mean (**D**) of T-Hg concentrations in hair for the sampling sites. SEM represents the standard error of the mean, and GM stands for geometric mean. The red line represents the maximum permissible hair T-Hg value recommended by the U.S. EPA [22]. *Statistical significance when comparing means against Arjona, with *p* < 0.05

**Fig. 3:**
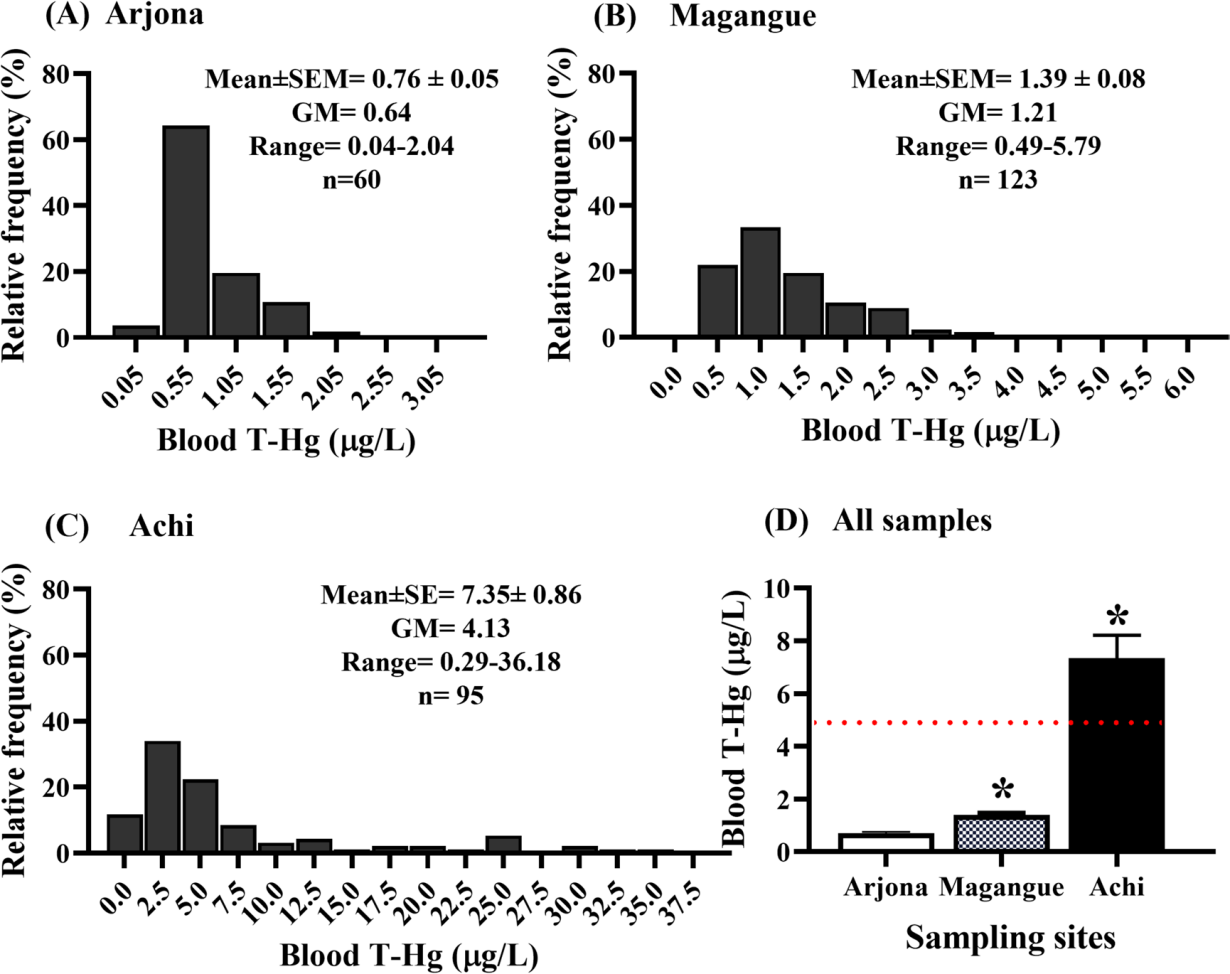
Frequency distribution (**A**–**C**) and mean (**D**) of T-Hg concentrations in blood for the sampling sites. SEM represents the standard error of the mean, and GM stands for geometric mean. The red line represents the maximum permissible blood T-Hg value established by INS [23]. *Statistical significance when comparing means against Arjona, with *p* < 0.05

The frequency distribution and means of T-Hg concentrations in hair and blood are presented in Fig. 2 and Fig. 3, respectively. The GM concentration of total hair mercury followed this order: Achi (0.97 µg/g) > Magangue (0.74 µg/g) > Arjona (0.23 µg/g) (Fig. 2). The same pattern was observed in blood, with Achi (4.13 µg/L) > Magangue (1.21 µg/L) > Arjona (0.64 µg/L) (Fig. 3). Children residing in Magangue and Achi had significantly higher mean Hg levels in their hair and blood compared to those of the children from Arjona children (*p* < 0.05).

Most children from Magangue (35.0%) and Achi (44.4%) had T-Hg concentrations in hair exceeded international guidelines (1 µg/g), while at the comparison site (Arjona), all samples were below 1 µg/g (mean 0.26 ± 0.02 µg/g, range 0.07–0.80 µg/g). Furthermore, 19.2% of the children from Achi presented concentrations higher than the provisional tolerable weekly intake (PTWI) proposed by the World Health Organization (WHO) (2.5 μg/g). GM values for Hg levels in hair were nearly 3- and fourfold greater in children from Magangue and Achi compared to those from Arjona (Fig. 2 and Fig. 3). Notably, only 4% of the children from Achi exhibited hair Hg concentrations > 5 µg/g. As for T-Hg in blood, children from Arjona had low levels (mean 0.76 ± 0.05 µg/L, range 0.04–2.04 µg/L), whereas in Achi and Magangue, 39.4 and 0.8% exhibited concentrations exceeding the international guidelines for blood Hg concentration (5 μg/L).

**Fig. 4:**
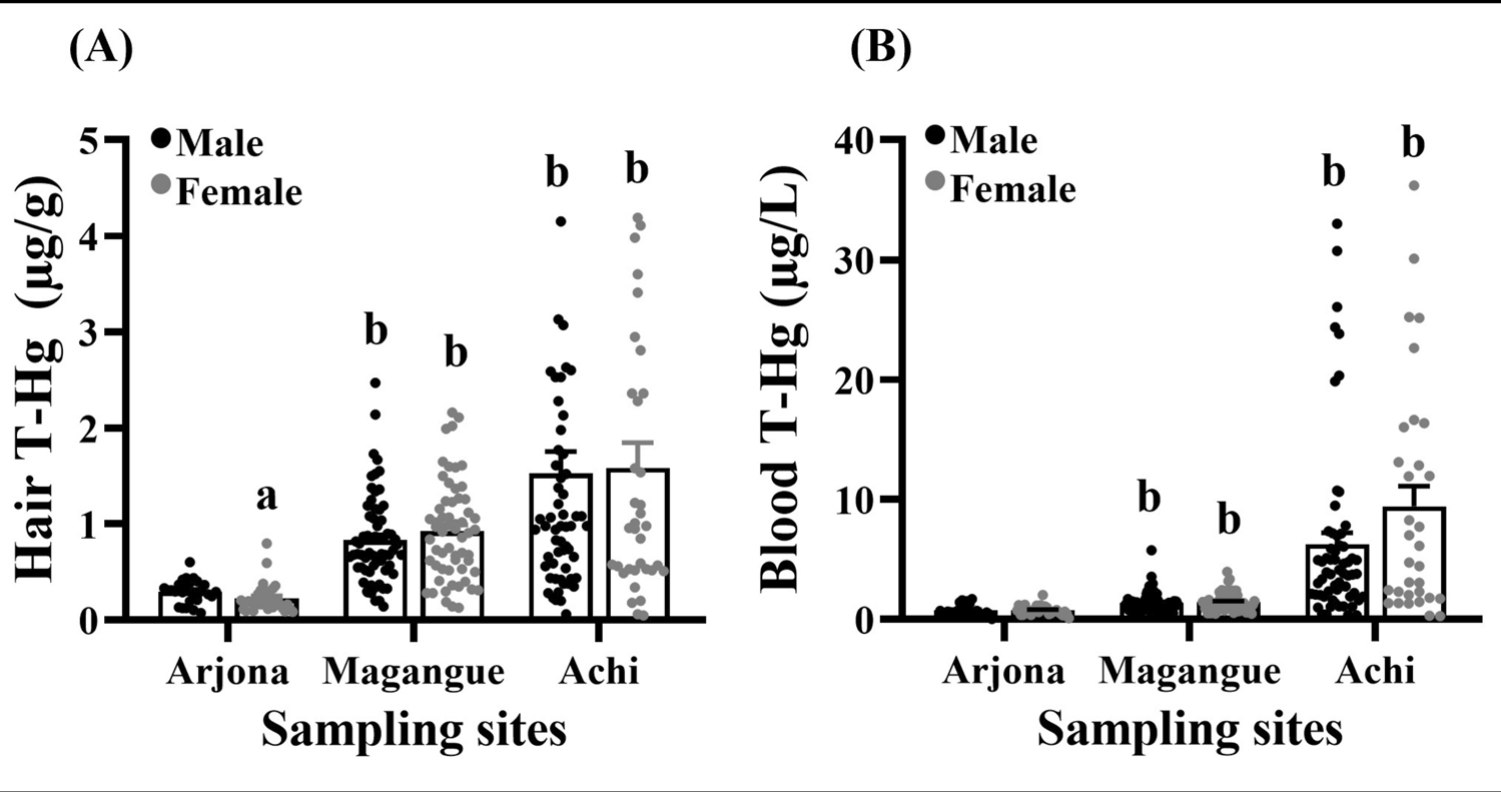
Comparison of mean T-Hg concentrations in hair (**A**) and blood (**B**) between sexes by sampling sites. Statistical significance when comparing means of males vs. females for the same site (a) and between sampling sites vs. Arjona for the same sex (b), *p* < 0.05

The mean distribution of Hg concentration in hair and blood by sex is illustrated in Fig. 4. No statistically significant differences were observed in hair or blood mercury levels between males and females from Magangue and Achi, but females and males from Arjona differ on their T-Hg in hair. In addition, when comparing mercury exposure in children of the same sex across the different sites in comparison to Arjona, significant differences were detected in Magangue and Achi.

Geometric means (GM) and percentiles of Hg levels in hair and blood for children from the sampling sites, according to variables such as sex, weight, and height, are presented in Supplementary Tables S3 and S4. The data followed a similar trend as the mean. It is worth noting that regarding weight and height, the highest concentrations of hair T-Hg were observed at the 95th percentile for children from Achi, with heights between 131 and 150 cm (7.4 µg/g) and weights between 26 and 30 kg (7.7 µg/g). The results were similar for blood T-Hg regarding weight, but the greater 95th percentile was found for children with height > 150 cm.

Spearman correlations for mercury levels in hair and blood are depicted in Supplementary Fig. S1. The results showed a very strong positive correlation between T-Hg concentration in blood and hair in Achi (*ρ* = 0.801; *p* < 0.01), a weak positive correlation in Magangue (*ρ* = 0.325; *p* < 0.01), and discernible relationship in Arjona. In addition, a moderate positive correlation (*ρ* = 0.437; *p* < 0.01) was found between fish consumption (meals/week) and blood T-Hg in Achi. For pooled data, fish consumption correlated better with T-Hg in hair (*ρ* = 0.276, *p* = 0.001) compared to blood (*ρ* = 0.144, *p* = 0.05) (Supplementary Table S5).

### Total Mercury Hair/Blood Ratio

**Fig. 5:**
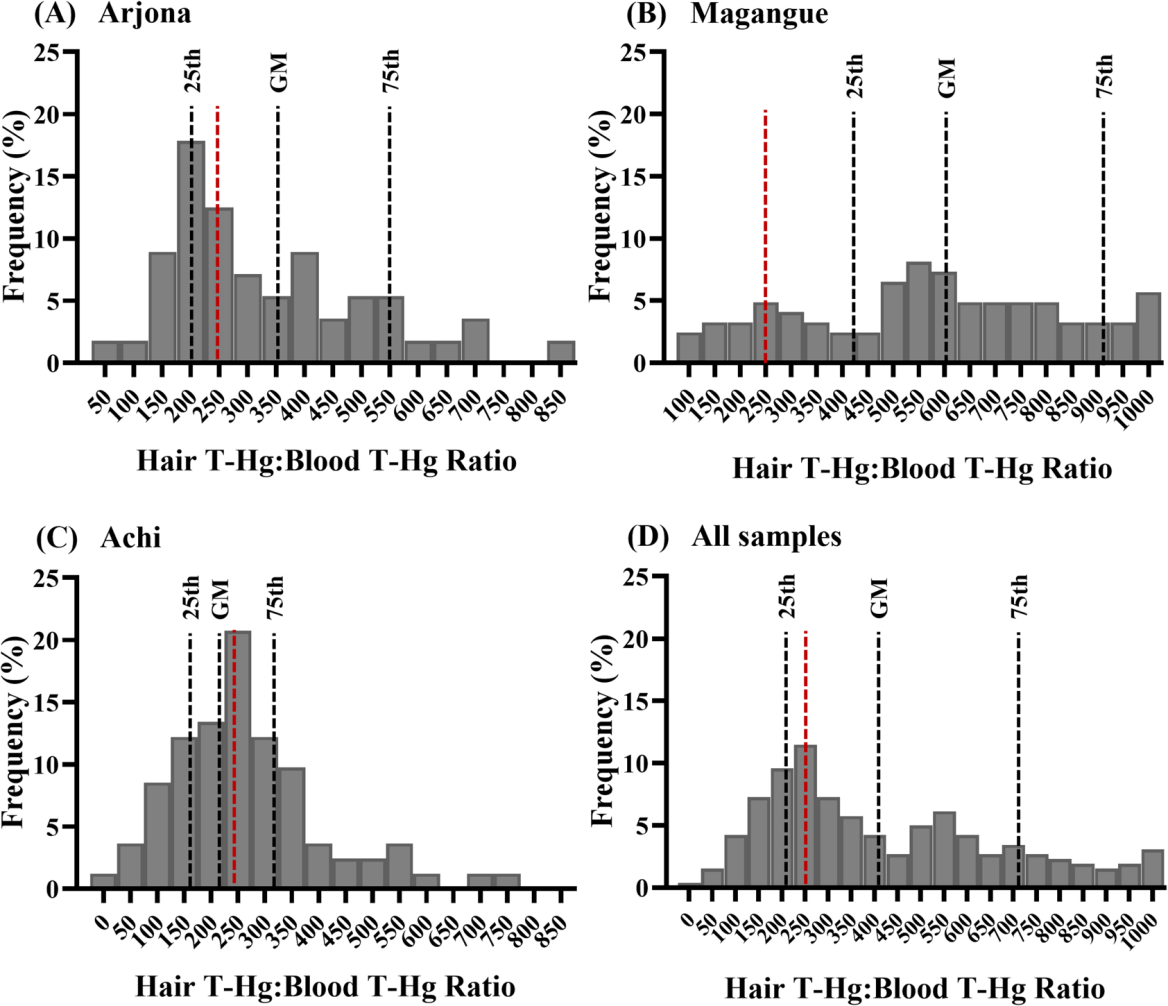
Histogram of T-Hg hair/blood ratio by sampling sites Arjona (**A**), Magangue (**B**), Achi (**C**), and all sites (**D**). The red line represents the reference value established by the WHO [47]

The distribution of T-Hg hair/blood ratios is depicted in Fig. 5. The GM for the entire child population was 402 and with an interquartile range (IQR) of 240–712, which was significantly higher than the 250:1 ratio determined by the WHO. However, variability among population subgroups was noted, with GM values of 359, 609, and 233 for Arjona, Magangue, and Achi, respectively.

### Hematological Parameters

Hematologic parameters and the frequency of values outside the reference ranges for different sampling sites are presented in Supplementary Table S6. Some significant mean differences were observed when children from Magangue and Achi were compared to Arjona. In particular, there were statistical differences observed in the mean values of hematological variables, including red blood cell parameters (HGB, MCH, MCHC, RDW), white blood cell parameters (WBC, GRA, LYM%, and GRA%), and platelet parameters (PLT, PCT, MPV, PDW) in Magangue. In Achi, the results varied among the parameters analyzed, with significant differences found for some red blood cell parameters, as well as differences in white blood cell parameters (WBC, LYM, GRA, and LYM%) and platelet parameters (MPV, PDW). Additionally, it was observed that 7.1% of children from Achi and 5% from Arjona had anemia based on hemoglobin levels.

Spearman’s correlations were examined between T-Hg concentrations in both hair and blood, and hematological parameters in children, stratified by sex (Tables 2 and 3). Total Hg levels in both hair and blood had distinct correlations with hematological variables when considering sampling sites. Correlations that reached significance for the entire population are displayed in Supplementary Fig. S2. Inverse correlations occurred for HGB, MCH, MCHC, and LYM%, and those positive were WBC, MCV, RDW, and GRA. Spearman correlations for all variables are presented in Supplementary Tables S7–10.

**Table 2:** Spearman’s correlation coefficient between hair T-Hg concentrations and hematological parameters in children by sex, across three sampling sites

Variable	Arjona, *n* = 60		Magangue, *n* = 123		Achi, *n* = 99	
	Male	Female	Male	Female	Male	Female
WBC (10^9^/L)	0.049 (0.404)a	− 0.284 (0.080)	0.001 (0.498)	0.255 (0.049)*	0.083 (0.281)	− 0.036 (0.428)
RBC (10^12^/L)	** −** 0.539 (0.002)*	0.192 (0.173)	− 0.157 (0.141)	0.083 (0.298)	0.027 (0.424)	− 0.155 (0.216)
HGB (g/dL)	** − **0.472 (0.007)*	− 0.043 (0.418)	− 0.069 (0.318)	0.069 (0.329)	− 0.009 (0.475)	0.063 (0.375)
HTC (%)	** − **0.558 (0.001)*	− 0.031 (0.440)	− 0.065 (0.329)	0.139 (0.186)	0.083 (0.281)	0.120 (0.271)
MCV (fL)	0.114 (0.286)	** − **0.422 (0.016)*	0.167 (0.126)	0.059 (0.353)	0.238 (0.045)*	0.228 (0.121)
MCH (pg)	0.395 (0.021)*	** − **0.433 (0.014)*	− 0.027 (0.427)	0.023 (0.443)	0.152 (0.141)	0.155 (0.216)
MCHC (g/L)	0.390 (0.022)*	− 0.089 (0.333)	− 0.194 (0.091)	− 0.204 (0.095)	− 0.143 (0.156)	0.027 (0.445)
PLT (10^9^/L)	0.137 (0.247)	0.189 (0.177)	0.337 (0.009)*	0.201 (0.099)	− 0.117 (0.206)	0.263 (0.088)
PCT (%)	0.118 (0.278)	0.073 (0.361)	0.395 (0.002)*	0.353 (0.010)*	− 0.014 (0.461)	0.315 (0.051)
MPV (fL)	− 0.208 (0.149)	0.070 (0.366)	− 0.014 (0.462)	0.076 (0.315)	0.233 (0.048)*	0.151 (0.222)
PDW (%)	− 0.173 (0.195)	0.206 (0.156)	− 0.036 (0.402)	0.144 (0.178)	0.136 (0.171)	0.019 (0.461)
RDW (%)	− 0.008 (0.485)	0.475 (0.010)*	− 0.094 (0.260)	− 0.197 (0.103)	− 0.025 (0.431)	− 0.255 (0.095)
LYM (10^9^/L)	− 0.010 (0.482)	0.137 (0.252)	− 0.161 (0.135)	0.249 (0.053)	− 0.031 (0.415)	− 0.155 (0.215)
GRA (10^9^/L)	0.019 (0.463)	− 0.299 (0.069)	0.117 (0.212)	0.174 (0.133)	0.120 (0.200)	0.028 (0.444)
LYM (%)	− 0.019 (0.462)	0.283 (0.081)	− 0.209 (0.075)	0.104 (0.254)	− 0.039 (0.392)	− 0.133 (0.250)
GRA (%)	0.085 (0.336)	− 0.247 (0.112)	0.201 (0.083)	0.025 (0.437)	0.125 (0.191)	0.115 (0.279)

^a^Data shown as Spearman’s correlation coefficient (p-value)

*Statistical significance (p <0.05)

**Table 3:** Spearman’s correlation coefficient between blood T-Hg concentrations and hematological parameters in children by sex, across three sampling sites

Variable	Arjona, *n* = 60		Magangue, *n* = 123		Achi, *n* = 99	
	Male	Female	Male	Female	Male	Female
WBC (10^9^/L)	0.094 (0.320)a	0.099 (0.319)	0.064 (0.335)	0.339 (0.013)*	0.116 (0.205)	0.179 (0.185)
RBC (10^12^/L)	0.096 (0.316)	0.245 (0.119)	− 0.026 (0.431)	0.099 (0.264)	− 0.167 (0.117)	0.372 (0.028)*
HGB (g/dL)	− 0.014 (0.472)	0.095 (0.325)	− 0.036 (0.401)	0.336 (0.014)*	0.049 (0.363)	− 0.001 (0.499)
HTC (%)	0.076 (0.353)	0.215 (0.151)	0.017 (0.455)	0.279 (0.035)*	0.049 (0.361)	0.093 (0.323)
MCV (fL)	− 0.146 (0.234)	− 0.081 (0.351)	0.122 (0.208)	0.209 (0.089)	0.387 (0.002)*	− 0.276 (0.082)
MCH (pg)	− 0.259 (0.096)	− 0.257 (0.108)	− 0.041 (0.392)	0.150 (0.168)	0.332 (0.008)*	** − **0.329 (0.047)*
MCHC (g/L)	− 0.044 (0.413)	− 0.325 (0.057)	** − **0.286 (0.026)*	− 0.162 (0.150)	− 0.039 (0.388)	− 0.217 (0.139)
PLT (10^9^/L)	0.411 (0.016)*	0.225 (0.139)	0.224 (0.065)	− 0.181 (0.123)	** − **0.266 (0.027)*	0.097 (0.315)
PCT (%)	0.375 (0.027)*	0.281 (0.087)	0.174 (0.113)	− 0.045 (0.386)	− 0.177 (0.102)	0.149 (0.229)
MPV (fL)	− 0.119 (0.276)	0.233 (0.132)	− 0.194 (0.096)	0.130 (0.202)	0.183 (0.095)	0.251 (0.103)
PDW (%)	− 0.114 (0.282)	0.149 (0.238)	− 0.231 (0.059)	0.102 (0.259)	0.123 (0.190)	0.038 (0.426)
RDW (%)	0.102 (0.303)	0.114 (0.294)	− 0.104 (0.243)	− 0.155 (0.159)	** − **0.229 (0.049)*	0.086 (0.336)
LYM (10^9^/L)	0.043 (0.413)	0.174 (0.203)	** − **0.249 (0.046)*	0.254 (0.050)	** − **0.255 (0.033)*	0.117 (0.280)
GRA (10^9^/L)	0.079 (0.345)	− 0.092 (0.332)	0.128 (0.196)	0.254 (0.050)	0.219 (0.057)	0.136 (0.249)
LYM (%)	0.074 (0.354)	0.090 (0.334)	** − **0.344 (0.009)*	0.057 (0.359)	** − **0.320 (0.009)*	− 0.069 (0.365)
GRA (%)	− 0.102 (0.303)	− 0.114 (0.295)	0.175 (0.119)	0.010 (0.484)	0.264 (0.028)*	0.158 (0.215)

^a^Data shown as Spearman’s correlation coefficient (p value)

*Statistical significance (p <0.05)

The results of multivariate relationships between the concentrations of T-Hg in hair and blood, age, sex, weight, height, and hematological parameters in the study population are presented in Supplementary Fig. S3. The two principal components (PCs) together accounted for approximately 29.1% of the variances. The primary source of variation, PC1, which represented 15.2% of the variance, showed strong positive loadings for GRA and GRA%, moderate positive loadings for age, WBC and RDWc, and weak positive loadings for hair T-Hg, blood T-Hg, RBC, and PLT. There were negative strong loadings for MCH, MCV, MCHC, and LYM%. The second component, PC2, represented 13.8% of the variance, in this, PC2 was observed strong positive loadings for age, height and weight, moderate positive loadings for HGB, MCV, MPV, PLT, LYM%, and PDWc (Supplementary Fig. S3). A visual examination of scatterplots of PC1 and PC2 scores revealed some overlapping dot clusters, particularly between Magangue and Achi in comparison to Arjona. Additionally, in Arjona, visual observation of plots of PC1 and PC2 scores showed variables grouped into two areas with moderate overlap between Magangue and Achi (Supplementary Fig. S3).

The results of the multiple linear regression analysis conducted to evaluate the hematological variables that showed a correlation with T-Hg concentrations in hair and blood are presented in Table 4. The regression model was also analyzed while controlling for potential confounding variables such as age, sex, and sampling location to specifically determine the associations between hematological parameters and T-Hg. The results obtained were statistically significant for WBC concentration (*β* = 0.053; *p* value = 0.021) and GRA (*β* = 0.086; *p* value = 0.011), establishing a positive association with T-Hg concentration in blood. Conversely, an inverse association was observed between lymphocyte percentage (LYM%) and T-Hg concentrations in blood (*β* =  − 0.353; *p* value = 0.036).

**Table 4:** Multiple linear regression analysis of T-Hg concentrations in hair and blood in relation to hematological parameters

Variables	Hair T-Hg (µg/g)				Blood T-Hg (µg/L)			
	Not adjusted		Adjusted		Not adjusted		Adjusted	
	β (*p* value)	95% CI	β (*p* value)	95% CI	β (*p* value)	95% CI	β (*p* value)	95% CI
WBC (10^9^/L)	0.079 (< 0.001)*	0.040; 0.117	0.024 (0.429)	− 0.036; 0.085	0.079 (< 0.001)*	0.046; 0.113	0.053 (0.021)*	0.007; 0.098
HGB (g/dL)	− 0.247 (< 0.001)*	− 0.358; − 0.137	0.069 (0.425)	− 0.240; 0.102	− 0.204 (< 0.001)*	− 0.302; − 0.107	− 0.025 (0.702)	− 0.153; 0.103
MCV (fL)	0.004 (0.187)	− 0.002; 0.011	0.002 (0.699)	− 0,007; 0.010	− 0.003 (0.333)	− 0.003; 0.009	− 0.0001 (0.998)	− 0.008; 0.008
MCH (pg)	− 0.363 (0.002)*	− 0.597; − 0.129	− 0.049 (0.791)	− 0.416; 0.317	− 0.409 (< 0.001)*	− 0.613; − 0.206	− 0.115 (0.408)	− 0.388; 0.158
MCHC (g/L)	− 0.615 (< 0.001)*	− 0.794; − 0.435	− 0.109 (0.391)	− 0.360; 0.141	− 0.626 (< 0.001)*	− 0.779; − 0.472	− 0.144 (0.132)	− 0.332; 0.044
RDWc (%)	0.015 (0.001)*	0.007; 0.022	− 0.001 (0.807)	− 0.013; 0.009	0.012 (< 0.001)*	0.006; 0.019	0.003 (0.482)	− 0.011; 0.005
MID (10^9^/L)	0.154 (0.001)*	0.063; 0.244	− 0.001 (0.983)	− 0.134; 0.131	0.142 (< 0.001)*	0.069; 0.214	− 0.033 (0.472)	− 0.125; 0.058
GRA (10^9^/L)	0.106 (< 0.001)*	0.051; 0.161	0.043 (0.338)	− 0.045; 0.131	0.089 (0.001)*	0.039; 0.139	0.086 (0.011)*	0.019; 0.152
LYM (%)	− 1.915 (0.005)*	− 3.247; − 0.582	− 0.582 (0.629)	− 2.647; 1.604	− 0.313 (0.013)*	− 0.561; − 0.066	− 0.353 (0.036)*	− 0.683; − 0.023
MID (%)	0.077 (0.069)	− 0.006; 0.161	− 0.026 (0.671)	− 0.149; 0.096	0.222 (< 0.001)*	0.145; 0.298	− 0.062 (0.627)	− 0.074; 0.122

Regression adjusted for age, sex, and sampling site.

CI, confidence interval.

*Statistical significance (*p* < 0.05)

## Discussion

In the Mojana region, located in the Bolivar department of Colombia, the municipalities of Magangue and Achi can be found along the banks of the Magdalena and Cauca rivers. These bodies of water are increasingly susceptible to Hg contamination due to gold mining activities taking place in the southern part of Bolívar department and the Cauca department. This situation raises significant concerns as children, especially those who are the offspring of fishermen residing along these riverbanks, are particularly vulnerable to exposure to this pollution. Despite regulatory efforts, the presence of informal mining operations and a lack of resources for enforcement continue to make the fight against contamination an ongoing challenge. The aforementioned concern has been substantiated through the findings in a recent study, where some individuals exhibited T-Hg levels in hair that surpassed the recommended maximum limit for adults, set at 1 µg/g. T-Hg levels in hair in children from Achi (1.55 μg/g, range = 0.05–8.83 μg/g) (Fig. 2) were similar to those found in adolescents aged 9 to 16 years (1.76 μg/g, range = 0.23–17.20) from Mojana region [48] and coastal populations in Colombia such as Tierrabomba (1.10 ± 0.07 μg/g) and San Onofre (1.87 ± 0.11 μg/g) for a group of participants aged between 11 and 18 years [31]. In addition, the mean hair Hg concentrations in children from Magangue (1.6-fold) and Achi (2.8-fold) were higher than those reported in First Nations adults (0.56 μg/g) [49].

The blood concentration of Hg serves as a critical indicator for assessing current and recent exposure, making it advisable to measure T-Hg in both hair and blood matrices to comprehensively assess exposure levels, potential risks, and potential associations with various sources and routes [50]. Regarding the results obtained in this study, blood T-Hg values reaching as high as 36.18 µg/L were observed, with the mean concentration showing significant variation depending on the geographical area, notably higher in Achi (7.31 µg/L), where fishing activities are more prominent, as well as the impact of gold mining on the Cauca River (Fig. 3). These findings surpass the blood Hg levels reported in other countries for children and adolescents potentially exposed through environmental factors or the consumption of fishery products [51, 52]. In comparison with studies conducted in mining regions within the country, the blood Hg levels were on average similar to those found in certain adult populations [53] and adolescents (4.11 µg/L) from Mojana region [48]. These results underscore the health risks faced by children residing in the areas under investigation. These values are particularly significant due to the vulnerability of children during these critical developmental phases, as low blood Hg concentrations have been linked to adverse health effects in children and adolescents [54]. The toxic effects of Hg exposure in children can manifest in the short, medium, or long term, contingent on the level of exposure and the specific developmental stage. Notably, exposure can even occur during fetal growth through the transfer of Hg via the maternal placenta's umbilical cord [28]. In this context, it has been observed that blood Hg concentrations lower than those observed in the municipality of Achi in this study have been associated with total cholesterol levels in adolescents [55]. Additionally, such concentrations can lead to disturbances in the central nervous system and neurodevelopmental disorders, such as autism spectrum disorder (ASD) [56, 57]. For instance, Hessabi, Rahbar [58] reported a mean blood Hg level of 0.35 μg/L in children with ASD, with 3.3% of the results exceeding 5 μg/L. In contrast, in the present study, 36% of apparently healthy children exhibited blood Hg concentrations exceeding the reference level [23].

Regarding comparing Hg concentrations in hair and blood, distinctions emerged between the study sites and sex, with females exhibiting the highest mean values (Fig. 4). In line with these findings, Batista, Schuhmacher [59] also noted in their study that females had higher hair T-Hg levels in schoolchildren aged 6–12 years. Furthermore, a more recent investigation underscored the influence of sex on hair metal concentrations among children residing in mining and industrial areas, where boys had lower Hg levels in their hair compared to females [60]. These disparities may be attributed to various factors, as proposed by Llop, Lopez-Espinosa [61] including differences in exposure patterns among the sexes, childhood behaviors, anatomical distinctions, variations in absorption mechanisms, and sex-specific metabolic conditions. These factors can enhance susceptibility to neurotoxic agents and potentially result in observable chemical effects, a notion corroborated by sex-specific studies on Hg toxicokinetics and other individual variables [62, 63].

In relation to the presence of Hg in children’s bodies and its potential sources, the exposure of preschoolers to Hg has been substantiated through the determination of total Hg in hair, coupled with its associations with the consumption of methylmercury-contaminated fish [64]. At both the national and international levels, the connection between human exposure to Hg and dietary habits, especially the consumption of fish products, has been well-established [4, 65–67]. In the present study, the consumption of fish was also evaluated, revealing that 96.1% of the population included in the study consume fish (Table 1). This high consumption rate can be attributed to the study area's location in a riverine zone, where fish serves as the primary food source. However, there were significant differences in consumption frequency between study sites, and a positive association was identified with total Hair T-Hg. While studies on children in this specific area are limited, scientific evidence elsewhere has indicated the presence of elevated concentrations of Hg and methylmercury in fish from the Colombian Caribbean, underscoring the potential risks associated with consumption [68].

In addition, for these communities, local fish and agricultural products are essential for their nutritional benefits, cost-effectiveness, and accessibility. This situation poses public health and food security concerns that require careful evaluation. In such contexts, assessing the proportion of Hg in hair and blood represents one of the most effective ways to gauge Hg exposure biomarkers [69]. The WHO has established a predefined ratio of 250:1, stipulating that Hg levels in hair should be approximately 250 times higher than in blood. In this study, the GM of Magangue significantly exceeded this predetermined value of 250, with considerable variability observed among the samples (interquartile range 445–938) (Fig. 5). It is important to note that such studies in children are relatively scarce. However, similar variability has been documented in other populations across various countries and subregions [69–72]. Thus, it is plausible that these ratios vary between sites due to demographic, biological, or dietary distinctions. Notably, Magangue is also a significant producer and consumer of rice. These disparities highlight the need for continued exploration of the Hg hair/Hg blood ratio to assess various populations and other relevant parameters.

Hematological parameters have been evaluated to provide insights into the health status and Hg exposure in children. The hematotoxicity of Hg in humans has not been entirely established. Animal studies have aided in identifying the mechanisms of action that affect red blood cells, white blood cells, and cells of the immune system [36, 37, 73–75]. Hematological parameters such HGB, HTC, RBC, WBC, MCV, MCH, and MCHC are commonly examined to assess the toxic stress induced by metals [37, 76]. In addition, abnormalities in RBC may be associated with hemolysis issues that can lead to severe conditions such as anemia, a common concern in children due to eating disorders, iron deficiency, or nutritional problems [77]. However, in recent years, hemolytic anemia has been linked to the presence of heavy metals in human blood, altering HGB concentrations [38]. Park and Lee [39] reported a positive relationship between Hg concentrations and HGB concentrations in a Korean adult population. Conversely, Weinhouse et al. [78] presented analyses from children (5–11 years) in artisanal gold mining communities in the Peruvian Amazon, where approximately 50% of the children were anemic, and T-Hg and HGB were inversely associated. These results are akin to those found in the present study (Tables 2 and 3), suggesting a potential link between Hg and methylmercury exposure from fish consumption and hemoglobin alterations that may lead to anemia in children in the Mojana region, demanding particular attention. Studies evaluating hematological changes in children due to Hg exposure are scarce. However, a study in coastal areas of the Colombian Caribbean indicated potential alterations in RBC and several abnormalities in WBC in adolescent children in areas potentially exposed to methylmercury through fish consumption, findings that could be compared with the present study [31].

In this study, a statistically significant positive association was observed through multiple linear regression analysis between blood T-Hg concentration and WBC and GRA, with a higher coefficient for GRA (Table 4). This could indicate a relationship between blood Hg levels and an increase in white blood cell concentration. Regarding this, some studies have explored the toxicological mechanisms of action that could be related to hematological and immune system alterations. Although a causal relationship has not been established in human studies, the positive association between Hg and GRA may be due to an inflammatory response to the presence of this toxic agent in the body. It is possible that this response is related to oxidative stress, inflammation, or autoimmunity induced by Hg [41]. In epidemiological studies, proinflammatory cytokines (IL-1β, TNF-α, IFN-γ, IL-6, IL-4, and IL-17) that stimulate the activation and production of neutrophils have been found and were positively associated with Hg exposure in mining and fishing populations of the Amazon [79, 80]. Likewise, immunostimulation and autoimmunity due to the activation of the innate immune system, mainly neutrophil granulocytes, have been established as a response to oxidative stress induced by ethyl-Hg into Hg^2+^, even at low concentrations [81]. The presence of heavy metals such as Hg in children’s bodies can increase the prevalence of diseases related to allergies, autoimmunity, and even cancer, by altering white blood cell concentrations and triggering inflammatory reactions [82].

In some cases, lymphocytes can also be affected by the presence of Hg and its organic forms, leading to immunostimulation and lymphoproliferation [83]. Similarly, the toxic forms of Hg can alter monocyte and LYM concentrations, in some scenarios leading to a positive correlation with T-Hg concentrations, as the entire hematological system may be sensitive to the presence of toxic substances. However, scientific evidence in this area remains limited [84]. However, positive correlations were found between the percentage of LYM and T-Hg in hair in adolescents from the Colombian Caribbean [31].

An opposite effect is immunosuppression caused by Hg and its organic species (MeHg) [81]. In this study, a statistically significant inverse association was observed between blood T-Hg concentration and LYM%, suggesting a possible impact of Hg exposure on the reduction of LYM%, which could also be related to an inflammatory response, possibly due to oxidative damage induced by Hg. A study in children associated prenatal MeHg exposure with a decrease in lymphocytes, including T cells, NK cells, B cells, as well as in monocytes [85]. In vitro studies have reported the effects of inorganic Hg compounds on lymphocyte markers, establishing a reduction due to apoptosis [86, 87]. Differences in responses reported by studies may be due to the type of research conducted, as human studies are scarce. Moreover, factors such as the characteristics of the studied population, exposure sources, exposure levels, and the evaluated forms of Hg may also influence the findings.

It is evident that Hg affects human health and should be considered a determining factor. However, further analysis of immunomarkers is needed to evaluate the mechanisms of action, effects, and preventive measures in populations, particularly in children who may be prone to severe diseases exacerbated by the presence of Hg in the body, even at low concentrations. Since each population is exposed to different environmental, social, economic, and genetic factors, there is a need to generate more scientific knowledge on Hg immunotoxicity to assess the health risks for the most vulnerable populations.

## Conclusions

Despite regulatory efforts, Hg continues to accumulate in the environment, leading to significant public health and food safety issues in areas with a high risk of exposure. This study reveals that children living along the banks of rivers contaminated by gold mining have T-Hg concentrations in blood and hair that may pose a potential health risk, with a large percentage exceeding the maximum permissible limit for humans. Notably, statistical analysis revealed a positive association between these T-Hg levels in blood and WBC and GRA concentrations, as well as an inverse association with LYM percentage. These findings support existing evidence linking Hg to toxicological mechanisms that trigger inflammatory responses and potential alterations in the immune system. Accordingly, further research is needed to evaluate specific biomarkers related to inflammation, oxidative stress, lymphocyte apoptosis, or endocrine disruption, which may be directly affected by Hg exposure, particularly in children. Since these populations may be more susceptible to infections and, consequently, experience a higher disease burden, safeguarding the health and well-being of children living in environmentally and socioeconomically vulnerable areas is essential.

Supplementary Information.

## Supplementary Information

Below is the link to the electronic supplementary material.

Supplementary file1 (DOCX 440 KB)

## Data Availability

No datasets were generated or analysed during the current study.
