# Supplementary material for: Hematological Parameters and Mercury Exposure in Children Living Along Gold-Mining-Impacted Rivers in the Mojana Region, Colombia
Source: Biol Trace Elem Res. 2025 Mar 1;203(10):5041–56. doi: 10.1007/s12011-025-04557-6 (PMC12507951; doi:10.1007/s12011-025-04557-6)
Supplement: Supplementary file 1 — Supplementary file1 (DOCX 440 KB) [file 12011_2025_4557_MOESM1_ESM.docx]

**Supplementary material**

**Hematological parameters and mercury exposure in children living along gold-mining-impacted rivers in the Mojana Region, Colombia**

Jenny Palomares-Bolaños^1,2^, Karina Caballero-Gallardo^1,2*^ and Jesus Olivero-Verbel^2^

**Authors Affiliations**

^1^ Functional Toxicology Group. School of Pharmaceutical Sciences. Zaragocilla Campus, University of Cartagena, Cartagena 130014, Colombia

^2^ Environmental and Computational Chemistry Group. School of Pharmaceutical Sciences, Zaragocilla Campus, University of Cartagena, Cartagena, 130014, Colombia

*Corresponding Author:

Prof. Karina Caballero Gallardo, Ph.D.

Functional Toxicology Group

School of Pharmaceutical Sciences

University of Cartagena

Cartagena, Colombia

Tel: 312-636-3365

Fax: 57-(5)-6699771

E-mail: kcaballerog@unicartagena.edu.co

**CONTENTS**

**I. Supplementary Figure 1 (Figure S1).** Spearman's correlation coefficient between hair T-Hg and blood T-Hg by sampling sites. The trendlines serve as additional information, showing that the data align more closely with a polynomial function of degree 2 than with a linear function.

**II. Supplementary Figure 2 (Figure S2).** Hematological parameters that exhibited a positive or negative Spearman correlation with T-Hg concentrations in both hair and blood for all participants.

**III. Supplementary Table 1 (Table S1).** General characteristics of the parents of the children from the different sampling sites.

**IV. Supplementary Table 2 (Table S2).** General characteristics during the early years of life.

**V. Supplementary Table 3 (Table S3).** Hair T-Hg concentrations in children's: Variations by sampling sites and percentile comparisons in relation to variables associated.

**VI. Supplementary Table 4 (Table S4).** Blood T-Hg concentrations in children's: Variations by sampling sites and percentile comparisons in relation to variables associated.

**VII. Supplementary Table 5 (Table S5).** Spearman's correlation between fish consumption and T-Hg concentrations in hair and blood at various sampling sites.

**VIII. Supplementary Table 6 (Table S6)**. Hematologic parameters in the blood and frequency of abnormal values by sampling site.

**IX. Supplementary Table 7 (Table S7).** Total correlation matrix for all variables included in the study.

**X. Supplementary Table 8 (Table S8).** Correlation matrix for Arjona with all variables included in the study.

**XI. Supplementary Table 9 (Table S9).** Correlation matrix for Magangue with all variables included in the study.

**XII. Supplementary Table 10 (Table S10).** Correlation matrix for Achi with all variables included in the study.

**XIII. Supplementary Figure S3.** Principal component analysis (PCA) for T-Hg concentrations in blood and hair, hematologic parameters, age, sex, and morphometric variables (A). Scatterplot of factor scores for PC1 and PC2 of analyzed variables across three sampling sites (B).


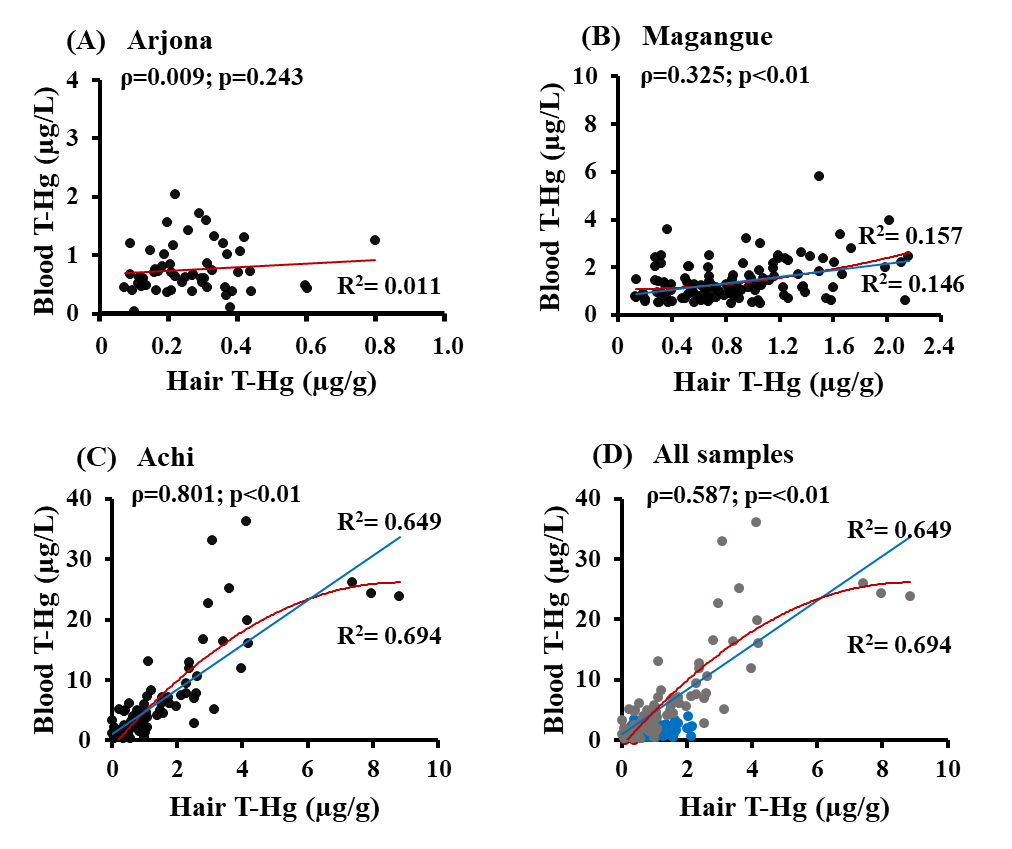


**I. Supplementary Figure S1.** Spearman's correlation coefficient between hair T-Hg and blood T-Hg by sampling sites. The trendlines serve as additional information, showing that the data align more closely with a polynomial function of degree 2 than with a linear function.


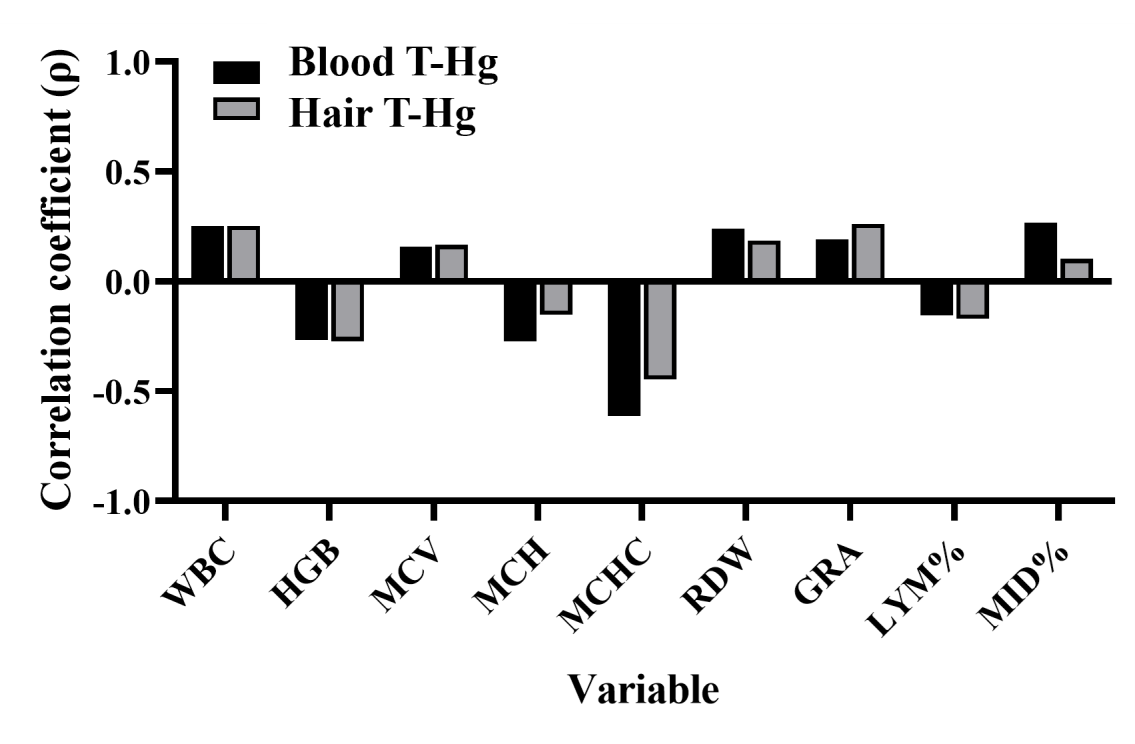


**II. Supplementary Figure S2.** Hematological parameters that exhibited a positive or negative Spearman correlation with T-Hg concentrations in both hair and blood for all participants.

**III. Supplementary Table S1.** General characteristics of the parents of the children from the different sampling sites.

| **Variable** | **Strata** |  | **Study area** | | | **Statistic** | ***p* value** |
| --- | --- | --- | --- | --- | --- | --- | --- |
|  |  | **Total n=282** | **Arjona n=60** | **Magangue n=123** | **Achi=99** |  |  |
| Age (years) | Mother  Mean±SEM  GM | 33.2±0.4  32.7 | 34.3±0.8  33.8 | 32.5±0.6  32.0 | 33.3±0.5  32.8 | KW= 4.2 | 0.12 |
|  | Father  Mean±SEM  GM | 38.8±0.5  38.1 | 37.6±0.8  37.1 | 39.1±0.9  38.2 | 39.4±0.8  38.6 | KW= 1.8 | 0.41 |
| Father occupation | Agriculture | 16 (5.7) | 2 (3.3) | 0 (0.0) | 14 (14.1) | *X^2^*=56.4 | <0.0001* |
|  | Fishing | 20 (7.1) | 0 (0.0) | 15 (12.2) | 5 (5.1) |  |  |
|  | Various occupations | 127 (45.0) | 21 (35) | 46 (37.4) | 60 (60.6) |  |  |
|  | Others (commerce, bricklaying, mechanics) | 119 (42.2) | 37 (61.7) | 62 (50.4) | 20 (20.2) |  |  |
| Time Word (years) | Mean±SEM  GM | 18.3±0.6  15.5 | 16.1±0.9  14.7 | 23.0±1.0  21.3 | 15.2±0.9  11.9 | KW= 32.3 | <0.0001* |
| Educational level | None | 69 (12.2) | 4 (3.3) | 42 (17.1) | 23 (11.6) | *X^2^=*101.4 | <0.0001* |
|  | Primary school | 170 (30.1) | 20 (16.7) | 93 (37.8) | 57 (28.8) |  |  |
|  | Secondary school | 273 (48.4) | 60 (50.0) | 108 (43.9) | 105 (53.0) |  |  |
|  | High school | 52 (9.3) | 36 (30.0) | 3 (1.2) | 13 (6.6) |  |  |
| Drug consumption | Yes | 9 (1.6) | 0 (0.0) | 7 (2.8) | 2 (1.0) | *X^2^*= 4.8 | 0.09 |
|  | No | 555 (98.4) | 120 (100.0) | 239 (97.2) | 196 (99.0) |  |  |
| Alcohol consumption | Yes | 227 (40.2) | 65 (54.2) | 111 (45.1) | 51 (25.8) | *X^2^*= 29.4 | <0.0001* |
|  | No | 337 (59.8) | 55 (45.8) | 135 (54.9) | 147 (74.2) |  |  |
| Tobacco consumption | Yes | 32(5.7) | 20 (16.7) | 11 (4.5) | 1 (0.5) | *X^2^*= 34.0 | <0.0001* |
|  | No | 542 (94.3) | 110 (83.3) | 235 (95.5) | 197 (99.5) |  |  |

The data are presented as the mean ± standard error of the mean or as frequencies n (%), depending on the variable.

KW=Kruskal-Wallis test followed by Dunn's multiple comparison test.

*X^2^*=Chi-square test.

Significance indicated by **p*<0.05.

**IV. Supplementary Table S2.** General characteristics during the early years of life.

| **Variable** | **Study area** | | | | | **Statistic** | ***p* value** |
| --- | --- | --- | --- | --- | --- | --- | --- |
|  | **Strata** | **Total n=282** | Arjona n=60 | Magangue n=123 | Achi=99 |  |  |
| Type of delivery | Cesarean | 147 (52.1) | 35 (58.3) | 67 (54.5) | 45 (45.5) | *X^2^*=2.9 | 0.23 |
|  | Vaginal delivery | 135 (47.9) | 25 (41.7) | 56 (45.5) | 54 (54.5) |  |  |
| Breast milk consumption | Yes | 257 (91.1) | 55 (91.7) | 112 (91.1) | 90 (90.9) | *X^2^*=0.03 | 0.99 |
|  | No | 25 (8.9) | 5 (8.3) | 11 (8.9) | 9 (9.1) |  |  |
| Premature delivery | Yes | 16 (5.7) | 2 (3.3) | 4 (3.3) | 10 (10.1) | *X^2^*=5.6 | 0.06 |
|  | No | 266 (94.3) | 58 (96.7) | 119 (96.7) | 89 (89.9) |  |  |

The data are presented as frequencies n (%).

*X^2^*=Chi-square test.

**V. Supplementary Table S3**. Hair T-Hg concentrations in children's: Variations by sampling sites and percentile comparisons in relation to variables associated.

| **Variable** | **Sampling sites** | **Arjona n=60**  **Hair T-Hg (µg/g)** | | | | | **Magangue n=123**  **Hair T-Hg (µg/g)** | | | | | **Achi=99**  **Hair T-Hg (µg/g)** | | | | |
| --- | --- | --- | --- | --- | --- | --- | --- | --- | --- | --- | --- | --- | --- | --- | --- | --- |
|  | **Category** | ***P25*** | ***P50*** | ***P75*** | ***P95*** | ***GM*** | ***P25*** | ***P50*** | ***P75*** | ***P95*** | ***GM*** | ***P25*** | ***P50*** | ***P75*** | ***P95*** | ***GM*** |
| *Sex* | Male | 0.2 | 0.3 | 0.4 | 0.5 | 0.3 | 0.5 | 0.7 | 1.1 | 1.6 | 0.7 | 0.5 | 1.0 | 1.8 | 7.4 | 1.0 |
|  | Female | 0.1 | 0.2 | 0.3 | 0.7 | 0.2 | 0.5 | 0.9 | 1.2 | 2.0 | 0.8 | 0.5 | 1.0 | 2.4 | 4.5 | 1.0 |
| *Weight (kg)* | 16-20 | 0.1 | 0.3 | 0.3 | 0.4 | 0.2 | 0.5 | 0.7 | 1.0 | 2.0 | 0.7 | 0.5 | 1.0 | 2.6 | 4.2 | 1.1 |
|  | 21-25 | 0.2 | 0.2 | 0.2 | 0.4 | 0.2 | 0.5 | 0.8 | 1.1 | 2.0 | 0.7 | 0.3 | 1.0 | 1.8 | 2.3 | 0.6 |
|  | 26-30 | 0.2 | 0.3 | 0.4 | 0.8 | 0.3 | 0.7 | 0.9 | 1.4 | 2.1 | 0.9 | 0.5 | 0.8 | 1.5 | 7.7 | 0.9 |
|  | >30 | 0.1 | 0.3 | 0.3 | 0.6 | 0.2 | 0.4 | 0.8 | 1.2 | 2.0 | 0.7 | 0.6 | 1.1 | 2.3 | 7.4 | 1.2 |
| *Height (cm)* | 100-130 | 0.2 | 0.2 | 0.3 | 0.4 | 0.2 | 0.5 | 0.7 | 1.1 | 1.8 | 0.7 | 0.5 | 1.0 | 2.2 | 4.7 | 0.9 |
|  | 131-150 | 0.1 | 0.3 | 0.4 | 0.7 | 0.3 | 0.6 | 0.8 | 1.2 | 2.0 | 0.8 | 0.5 | 1.0 | 2.3 | 7.4 | 1.0 |
|  | >150 | 0.1 | 0.3 | 0.3 | 0.4 | 0.2 | 0.4 | 0.7 | 1.0 | 1.2 | 0.6 | 0.5 | 1.0 | 1.8 | 2.8 | 1.0 |

P25, P50, P75, P95= Percentiles.

GM: Geometric mean.

**VI. Supplementary Table S4.** Blood T-Hg concentrations in children's: Variations by sampling sites and percentile comparisons in relation to variables associated.

| **Variable** | **Sampling sites** | **Arjona n=60**  **Blood T-Hg (µg/L)** | | | | | **Magangue n=123**  **Blood T-Hg (µg/L)** | | | | | **Achi=95**  **Blood T-Hg (µg/L)** | | | | |
| --- | --- | --- | --- | --- | --- | --- | --- | --- | --- | --- | --- | --- | --- | --- | --- | --- |
|  | **Category** | ***P25*** | ***P50*** | ***P75*** | ***P95*** | ***GM*** | ***P25*** | ***P50*** | ***P75*** | ***P95*** | ***GM*** | ***P25*** | ***P50*** | ***P75*** | ***P95*** | ***GM*** |
| Sex | Male | 0.2 | 0.6 | 1.1 | 1.7 | 0.6 | 0.5 | 1.1 | 1.6 | 2.9 | 1.2 | 1.9 | 3.9 | 6.8 | 26.0 | 3.8 |
|  | Female | 0.1 | 0.7 | 1.0 | 1.7 | 0.7 | 0.5 | 1.2 | 2.0 | 3.2 | 1.2 | 2.1 | 5.5 | 13.9 | 31.6 | 5.3 |
| Weight (kg) | 16-20 | 0.5 | 0.7 | 1.1 | 1.2 | 0.7 | 0.8 | 1.1 | 1.5 | 2.0 | 1.1 | 1.6 | 3.7 | 13.3 | 22.7 | 4.1 |
|  | 21-25 | 0.5 | 0.6 | 0.8 | 2.0 | 0.6 | 0.7 | 1.0 | 1.5 | 2.6 | 1.1 | 2.1 | 4.2 | 7.4 | 20.3 | 3.8 |
|  | 26-30 | 0.4 | 0.6 | 1.2 | 1.6 | 0.6 | 0.7 | 1.4 | 2.1 | 3.6 | 1.3 | 1.5 | 3.4 | 7.9 | 31.8 | 3.7 |
|  | >30 | 0.4 | 0.6 | 0.9 | 1.7 | 0.6 | 0.9 | 1.2 | 2.2 | 3.5 | 1.3 | 2.2 | 5.0 | 10.7 | 30.6 | 4.8 |
| Height (cm) | 100-130 | 0.5 | 0.7 | 1.0 | 1.9 | 0.7 | 0.7 | 1.1 | 1.6 | 2.3 | 1.1 | 2.1 | 4.5 | 9.5 | 25.9 | 4.4 |
|  | 131-150 | 0.5 | 0.6 | 0.9 | 1.4 | 0.6 | 0.8 | 1.2 | 2.2 | 3.5 | 1.3 | 1.8 | 4.0 | 7.8 | 28.1 | 4.0 |
|  | >150 | 0.4 | 0.7 | 1.6 | 1.7 | 0.8 | 0.9 | 1.1 | 2.4 | 2.5 | 1.3 | 2.2 | 2.7 | 12.3 | 30.7 | 4.3 |

P25, P50, P75, P95= Percentiles.

GM: Geometric mean.

**VII. Supplementary Table S5.** Spearman's correlation between fish consumption and T-Hg concentrations in hair and blood at various sampling sites.

|  | **Hair T-Hg (µg/g)** | | | | **Blood T-Hg (µg/L)** | | | |
| --- | --- | --- | --- | --- | --- | --- | --- | --- |
| **Variable** | **Arjona n=46** | **Magangue n=55** | **Achi n=44** | **All participants** | **Arjona n=46** | **Magangue n=55** | **Achi n=44** | **All participants** |
| Fish consumption (meals/week) | 0.003 (0.492) | -0.008 (0.476) | 0.101 (0.282) | **0.276 (0.001)** | **-0.282 (0.034)** | 0.015 (0.455) | **0.437 (0.004)** | 0.144 (0.05) |

The data is presented as Spearman's correlation coefficient (*p* value). Statistical significance is indicated by bold letters (*p* < 0.05).

**VIII. Supplementary Table S6.** Hematologic parameters in the blood and frequency of abnormal values by sampling site.

| **Variable** | **Unit** | **Reference value** | **Arjona n=60** | | | **Magangue n=123** | | | **Achi n=99** | | |
| --- | --- | --- | --- | --- | --- | --- | --- | --- | --- | --- | --- |
|  |  |  | **Mean ± standard error** | **Below reference value (%)** | **Above reference value (%)** | **Mean ± standard error** | **Below reference value (%)** | **Above reference value (%)** | **Mean ± standard error** | **Below reference value (%)** | **Above reference value (%)** |
| *Red blood cell* | | | | | | | | | | | |
| RBC | 10^12^/L | 4.1-5.6 | 4.7 ± 0.03 | 1.7 | 0.0 | 4.7 ± 0.04 | 1.6 | 0.8 | 4.7 ± 0.04 | 2.0 | 0.0 |
| HGB | g/dL | 11.5-16.3 | 13.3 ± 0.1 | 5.0 | 0.0 | **12.8 ± 0.1*** | 1.6 | 0.0 | **12.4 ± 0.1*** | 7.1 | 0.0 |
| HTC | % | 34.4-48.3 | 37.6 ± 0.3 | 16.7 | 0.0 | 37.4 ± 0.3 | 6.5 | 0.8 | 37.4 ± 0.2 | 6.1 | 0.0 |
| MCV | fL | 74.3-93 | 79.8 ± 0.4 | 16.7 | 0.0 | 80.2 ± 0.3 | 3.3 | 0.0 | 80.1 ± 0.5 | 6.1 | 0.0 |
| MCH | pg | 24.3-31.7 | 28.2 ± 0.1 | 1.7 | 0.0 | **27.6 ± 0.1*** | 0.8 | 0.0 | **26.6 ± 0.2*** | 6.1 | 1.0 |
| MCHC | g/dL | 31.9-35.1 | 35.4 ± 0.1 | 5.0 | 46.7 | **34.4 ± 0.1*** | 0.8 | 15.4 | **33.2 ± 0.1*** | 1.0 | 1.0 |
| RDW | % | 11.7-14.3 | 16.1 ± 0.1 | 0.0 | 0.0 | **16.8 ± 0.1*** | 0.0 | 5.7 | **17.3 ± 0.1*** | 0.0 | 7.0 |
| *White blood cell* | | | | | | | | | | | |
| WBC | 10^9^/L | 3.7-11.9 | 6.8 ± 0.2 | 1.7 | 0.0 | **8.4 ± 0.2*** | 0.0 | 4.9 | **8.9 ± 0.0*** | 0.0 | 10.1 |
| LYM | 10^9^/L | 2.2-6.8 | 2.9 ± 0.1 | 10.0 | 0.0 | 3.0 ± 0.1 | 9.8 | 0.0 | **3.2 ± 0.1*** | 3.0 | 2.0 |
| GRA | 10^9^/L | 2.5-7.5 | 3.5 ± 0.1 | 15.0 | 0.0 | **4.9 ± 0.2*** | 1.6 | 5.7 | **4.7 ± 0.2*** | 11.1 | 7.1 |
| LYM | % | 20-56.6 | 42.8 ± 1.1 | 1.7 | 10.0 | **35.9 ± 0.8*** | 2.4 | 0.0 | **37.7 ± 1.0*** | 1.0 | 1.0 |
| GRA | % | 33.2-74.7 | 50.2 ± 1.1 | 6.7 | 1.7 | **57.7 ± 0.9*** | 0.0 | 2.4 | 51.7 ± 1.1 | 3.0 | 1.0 |
| *Platelet* | | | | | | | | | | | |
| PLT | 10^9^/L | 194-477 | 297.4 ± 5.9 | 1.7 | 1.7 | **318.5 ± 7.8*** | 4.1 | 2.4 | 287.0 ± 6.6 | 4.0 | 0.0 |
| PCT | % | 0.1-0.5 | 0.2 ± 0.004 | 0.0 | 0.0 | 0.2 ± 0.01 | 0.0 | 0.0 | 0.2 ± 0.01 | 0.0 | 0.0 |
| MPV | fL | 6.6-9.9 | 8.2 ± 0.1 | 0.0 | 1.7 | **7.8 ± 0.1*** | 2.4 | 1.6 | **7.8 ± 0.1*** | 3.0 | 3.0 |
| PDW | % | 25-50 | 38.8 ± 0.1 | 0.0 | 0.0 | **38.2 ± 0.2*** | 0.0 | 0.0 | **38.0 ± 0.2*** | 0.0 | 0.0 |

* Statistical significance (*p* < 0.05).

Abbreviations: WBC, white blood cell count; RBC, red blood cell count; HGB, hemoglobin concentration; HTC, hematocrit; MCV, mean corpuscular volume; MCH, mean corpuscular hemoglobin; MCHC, mean corpuscular hemoglobin concentration; PLT, total platelet count; PCT, plateletcrit; MPV, mean platelet volume; PDW, platelet distribution width; RDW, red blood cell distribution width; LYM, lymphocyte count; GRA, granulocyte count; LYM%, lymphocyte percentage; GRA%, granulocyte percentage.

**IX. Supplementary Table S7.** Total correlation matrix for all variables included in the study.

| **Variable** | **Blood**  **T-Hg** | **Hair**  **T-Hg** | **WBC** | **RCB** | **HGB** | **HTC** | **MCV** | **MCH** | **MCHC** | **PLT** | **PCT** | **MPV** | **PDW** | **RDW** | **LYM** | **MID** | **GRA** | **LYM%** | **MID%** | **GRA%** | **Sex** | **Age** | **Weight** | **Height** | **BMI** |
| --- | --- | --- | --- | --- | --- | --- | --- | --- | --- | --- | --- | --- | --- | --- | --- | --- | --- | --- | --- | --- | --- | --- | --- | --- | --- |
| **Blood**  **T-Hg** | 1.000 |  |  |  |  |  |  |  |  |  |  |  |  |  |  |  |  |  |  |  |  |  |  |  |  |
| **Hair**  **T-Hg** | 0.603 | 1.000 |  |  |  |  |  |  |  |  |  |  |  |  |  |  |  |  |  |  |  |  |  |  |  |
| **WBC** | 0.253 | 0.252 | 1.000 |  |  |  |  |  |  |  |  |  |  |  |  |  |  |  |  |  |  |  |  |  |  |
| **RCB** | -0.020 | -0.139 | 0.004 | 1.000 |  |  |  |  |  |  |  |  |  |  |  |  |  |  |  |  |  |  |  |  |  |
| **HGB** | -0.266 | -0.272 | -0.020 | 0.567 | 1.000 |  |  |  |  |  |  |  |  |  |  |  |  |  |  |  |  |  |  |  |  |
| **HTC** | 0.075 | 0.004 | 0.076 | 0.702 | 0.789 | 1.000 |  |  |  |  |  |  |  |  |  |  |  |  |  |  |  |  |  |  |  |
| **MCV** | 0.160 | 0.166 | 0.081 | -0.460 | 0.191 | 0.209 | 1.000 |  |  |  |  |  |  |  |  |  |  |  |  |  |  |  |  |  |  |
| **MCH** | -0.274 | -0.152 | -0.022 | -0.484 | 0.366 | 0.032 | 0.758 | 1.000 |  |  |  |  |  |  |  |  |  |  |  |  |  |  |  |  |  |
| **MCHC** | -0.613 | -0.448 | -0.132 | -0.186 | 0.361 | -0.210 | -0.025 | 0.583 | 1.000 |  |  |  |  |  |  |  |  |  |  |  |  |  |  |  |  |
| **PLT** | -0.086 | 0.063 | 0.089 | 0.004 | -0.046 | -0.067 | -0.096 | -0.022 | 0.076 | 1.000 |  |  |  |  |  |  |  |  |  |  |  |  |  |  |  |
| **PCT** | -0.113 | 0.054 | 0.131 | 0.032 | 0.029 | -0.019 | -0.062 | 0.029 | 0.125 | 0.851 | 1.000 |  |  |  |  |  |  |  |  |  |  |  |  |  |  |
| **MPV** | -0.063 | -0.074 | 0.019 | 0.069 | 0.179 | 0.118 | 0.070 | 0.109 | 0.104 | -0.447 | 0.026 | 1.000 |  |  |  |  |  |  |  |  |  |  |  |  |  |
| **PDW** | -0.114 | -0.098 | 0.011 | 0.045 | 0.167 | 0.094 | 0.058 | 0.118 | 0.121 | -0.382 | 0.059 | 0.927 | 1.000 |  |  |  |  |  |  |  |  |  |  |  |  |
| **RDW** | 0.240 | 0.185 | 0.050 | 0.314 | -0.217 | -0.015 | -0.489 | -0.616 | -0.379 | 0.079 | 0.009 | -0.177 | -0.169 | 1.000 |  |  |  |  |  |  |  |  |  |  |  |
| **LYM** | 0.097 | 0.073 | 0.498 | 0.008 | 0.006 | 0.039 | 0.027 | -0.009 | -0.054 | -0.028 | 0.036 | 0.076 | 0.050 | -0.077 | 1.000 |  |  |  |  |  |  |  |  |  |  |
| **MID** | 0.373 | 0.220 | 0.425 | -0.055 | -0.139 | 0.047 | 0.121 | -0.088 | -0.324 | -0.098 | -0.070 | 0.008 | -0.018 | 0.144 | 0.270 | 1.000 |  |  |  |  |  |  |  |  |  |
| **GRA** | 0.193 | 0.263 | 0.876 | 0.026 | 0.002 | 0.081 | 0.066 | -0.013 | -0.102 | 0.157 | 0.178 | -0.011 | -0.004 | 0.111 | 0.130 | 0.192 | 1.000 |  |  |  |  |  |  |  |  |
| **LYM%** | -0.154 | -0.170 | -0.439 | -0.032 | 0.023 | -0.069 | -0.032 | 0.044 | 0.111 | -0.136 | -0.124 | 0.041 | 0.012 | -0.146 | 0.485 | -0.176 | -0.722 | 1.000 |  |  |  |  |  |  |  |
| **MID%** | 0.265 | 0.103 | -0.013 | -0.070 | -0.130 | 0.010 | 0.101 | -0.061 | -0.266 | -0.190 | -0.177 | 0.009 | -0.017 | 0.111 | 0.034 | 0.869 | -0.216 | 0.018 | 1.000 |  |  |  |  |  |  |
| **GRA%** | 0.025 | 0.154 | 0.383 | 0.036 | 0.021 | 0.042 | 0.011 | 0.008 | 0.009 | 0.213 | 0.179 | -0.070 | -0.035 | 0.117 | -0.432 | -0.184 | 0.743 | -0.876 | -0.398 | 1.000 |  |  |  |  |  |
| **Sex** | 0.077 | 0.039 | -0.050 | 0.027 | -0.117 | -0.059 | -0.144 | -0.176 | -0.116 | -0.047 | -0.032 | 0.054 | 0.075 | 0.143 | -0.066 | 0.016 | -0.014 | -0.040 | 0.051 | 0.011 | 1.000 |  |  |  |  |
| **Age** | 0.092 | 0.090 | -0.085 | 0.058 | 0.104 | 0.177 | 0.109 | 0.065 | -0.046 | -0.118 | -0.091 | 0.049 | 0.064 | 0.077 | -0.182 | -0.006 | 0.020 | -0.127 | 0.046 | 0.139 | 0.103 | 1.000 |  |  |  |
| **Weight** | 0.084 | 0.043 | -0.062 | 0.102 | 0.152 | 0.237 | 0.139 | 0.065 | -0.089 | -0.178 | -0.114 | 0.171 | 0.146 | 0.039 | -0.129 | 0.025 | 0.030 | -0.094 | 0.068 | 0.083 | 0.064 | 0.775 | 1.000 |  |  |
| **Height** | 0.050 | -0.007 | -0.101 | 0.062 | 0.132 | 0.183 | 0.136 | 0.099 | -0.021 | -0.138 | -0.080 | 0.143 | 0.146 | -0.007 | -0.200 | 0.038 | 0.007 | -0.149 | 0.095 | 0.116 | 0.087 | 0.855 | 0.879 | 1.000 |  |
| **BMI** | 0.089 | 0.094 | 0.004 | 0.115 | 0.098 | 0.199 | 0.083 | -0.021 | -0.156 | -0.207 | -0.148 | 0.176 | 0.134 | 0.088 | 0.024 | -0.004 | 0.028 | 0.026 | 0.001 | -0.004 | 0.004 | 0.369 | 0.765 | 0.395 | 1.000 |

The Spearman correlation coefficient is shown. Values with statistical significance are highlighted in red. Blood T-Hg: µg/L; Hair T-Hg: µg/g; Age: years; Weight: kg; Height: cm, and BMI: Body Mass Index.

**X. Supplementary Table S8.** Correlation matrix for Arjona with all variables included in the study.

| **Variable** | **Blood**  **T-Hg** | **Hair**  **T-Hg** | **WBC** | **RCB** | **HGB** | **HTC** | **MCV** | **MCH** | **MCHC** | **PLT** | **PCT** | **MPV** | **PDW** | **RDW** | **LYM** | **MID** | **GRA** | **LYM%** | **MID%** | **GRA%** | **Sex** | **Age** | **Weight** | **Height** | **BMI** |
| --- | --- | --- | --- | --- | --- | --- | --- | --- | --- | --- | --- | --- | --- | --- | --- | --- | --- | --- | --- | --- | --- | --- | --- | --- | --- |
| **Blood**  **T-Hg** | 1.000 |  |  |  |  |  |  |  |  |  |  |  |  |  |  |  |  |  |  |  |  |  |  |  |  |
| **Hair**  **T-Hg** | 0.140 | 1.000 |  |  |  |  |  |  |  |  |  |  |  |  |  |  |  |  |  |  |  |  |  |  |  |
| **WBC** | 0.045 | -0.123 | 1.000 |  |  |  |  |  |  |  |  |  |  |  |  |  |  |  |  |  |  |  |  |  |  |
| **RCB** | 0.142 | -0.203 | 0.024 | 1.000 |  |  |  |  |  |  |  |  |  |  |  |  |  |  |  |  |  |  |  |  |  |
| **HGB** | -0.048 | -0.259 | -0.032 | 0.684 | 1.000 |  |  |  |  |  |  |  |  |  |  |  |  |  |  |  |  |  |  |  |  |
| **HTC** | 0.075 | -0.322 | 0.000 | 0.758 | 0.918 | 1.000 |  |  |  |  |  |  |  |  |  |  |  |  |  |  |  |  |  |  |  |
| **MCV** | -0.144 | -0.170 | -0.097 | -0.287 | 0.273 | 0.294 | 1.000 |  |  |  |  |  |  |  |  |  |  |  |  |  |  |  |  |  |  |
| **MCH** | -0.294 | -0.017 | -0.069 | -0.491 | 0.159 | 0.013 | 0.839 | 1.000 |  |  |  |  |  |  |  |  |  |  |  |  |  |  |  |  |  |
| **MCHC** | -0.151 | 0.282 | 0.104 | -0.502 | -0.307 | -0.578 | -0.147 | 0.285 | 1.000 |  |  |  |  |  |  |  |  |  |  |  |  |  |  |  |  |
| **PLT** | 0.229 | 0.171 | 0.068 | -0.038 | 0.022 | -0.006 | 0.017 | 0.077 | 0.096 | 1.000 |  |  |  |  |  |  |  |  |  |  |  |  |  |  |  |
| **PCT** | 0.252 | 0.137 | 0.126 | 0.051 | 0.044 | 0.046 | 0.051 | 0.081 | 0.049 | 0.890 | 1.000 |  |  |  |  |  |  |  |  |  |  |  |  |  |  |
| **MPV** | 0.067 | -0.042 | 0.186 | 0.189 | -0.026 | 0.036 | -0.076 | -0.146 | -0.093 | -0.450 | -0.053 | 1.000 |  |  |  |  |  |  |  |  |  |  |  |  |  |
| **PDW** | 0.032 | 0.025 | 0.147 | 0.147 | -0.057 | 0.036 | -0.094 | -0.131 | -0.178 | -0.350 | -0.015 | 0.860 | 1.000 |  |  |  |  |  |  |  |  |  |  |  |  |
| **RDW** | 0.066 | 0.274 | -0.033 | 0.284 | 0.068 | -0.057 | -0.571 | -0.383 | 0.193 | 0.248 | 0.277 | 0.007 | 0.044 | 1.000 |  |  |  |  |  |  |  |  |  |  |  |
| **LYM** | 0.065 | 0.056 | 0.537 | -0.036 | -0.063 | -0.040 | -0.098 | -0.082 | 0.113 | 0.106 | 0.085 | -0.024 | -0.106 | -0.090 | 1.000 |  |  |  |  |  |  |  |  |  |  |
| **MID** | 0.051 | -0.031 | 0.428 | 0.059 | 0.200 | 0.132 | 0.059 | 0.136 | 0.117 | -0.052 | -0.005 | 0.097 | 0.099 | -0.013 | 0.110 | 1.000 |  |  |  |  |  |  |  |  |  |
| **GRA** | -0.079 | -0.120 | 0.821 | 0.057 | 0.025 | 0.062 | -0.066 | -0.053 | -0.005 | 0.048 | 0.102 | 0.208 | 0.245 | -0.020 | 0.062 | 0.335 | 1.000 |  |  |  |  |  |  |  |  |
| **LYM%** | 0.124 | 0.125 | -0.285 | -0.098 | -0.093 | -0.092 | -0.005 | -0.049 | 0.057 | 0.013 | -0.040 | -0.178 | -0.273 | -0.095 | 0.586 | -0.306 | -0.727 | 1.000 |  |  |  |  |  |  |  |
| **MID%** | -0.041 | 0.011 | -0.231 | 0.059 | 0.271 | 0.143 | 0.125 | 0.228 | 0.055 | -0.115 | -0.111 | -0.030 | 0.009 | 0.096 | -0.306 | 0.730 | -0.197 | -0.175 | 1.000 |  |  |  |  |  |  |
| **GRA%** | -0.168 | -0.102 | 0.328 | 0.014 | 0.051 | -0.006 | -0.021 | 0.081 | -0.006 | 0.056 | 0.076 | 0.116 | 0.185 | 0.157 | -0.489 | 0.177 | 0.733 | -0.929 | 0.063 | 1.000 |  |  |  |  |  |
| **Sex** | 0.039 | 0.365 | -0.165 | -0.083 | 0.021 | -0.027 | -0.021 | 0.068 | 0.142 | 0.004 | 0.017 | 0.088 | 0.146 | 0.165 | -0.185 | 0.007 | -0.020 | -0.131 | 0.125 | 0.065 | 1.000 |  |  |  |  |
| **Age** | -0.007 | 0.069 | -0.067 | 0.232 | 0.248 | 0.176 | -0.133 | -0.069 | -0.049 | -0.120 | -0.143 | 0.073 | 0.060 | 0.171 | -0.255 | -0.019 | 0.094 | -0.237 | 0.121 | 0.266 | 0.219 | 1.000 |  |  |  |
| **Weight** | -0.024 | 0.129 | -0.180 | 0.207 | 0.269 | 0.216 | -0.036 | -0.042 | -0.056 | -0.133 | -0.112 | 0.164 | 0.162 | 0.065 | -0.262 | -0.026 | -0.002 | -0.140 | 0.121 | 0.124 | 0.146 | 0.803 | 1.000 |  |  |
| **Height** | 0.059 | 0.065 | -0.251 | 0.248 | 0.279 | 0.257 | -0.048 | -0.056 | -0.171 | -0.151 | -0.172 | 0.049 | 0.113 | 0.066 | -0.340 | -0.066 | -0.040 | -0.178 | 0.144 | 0.162 | 0.184 | 0.912 | 0.845 | 1.000 |  |
| **BMI** | -0.088 | 0.102 | -0.019 | 0.106 | 0.134 | 0.062 | -0.057 | -0.058 | 0.105 | -0.121 | -0.059 | 0.226 | 0.131 | 0.050 | 0.002 | 0.014 | 0.001 | 0.038 | 0.015 | -0.050 | -0.066 | 0.264 | 0.691 | 0.242 | 1.000 |

The Spearman correlation coefficient is shown. Values with statistical significance are highlighted in red. Blood T-Hg: µg/L; Hair T-Hg: µg/g; Age: years; Weight: kg; Height: cm, and BMI: Body Mass Index.

**XI. Supplementary Table S9.** Correlation matrix for Magangue with all variables included in the study.

| **Variable** | **Blood**  **T-Hg** | **Hair**  **T-Hg** | **WBC** | **RCB** | **HGB** | **HTC** | **MCV** | **MCH** | **MCHC** | **PLT** | **PCT** | **MPV** | **PDW** | **RDW** | **LYM** | **MID** | **GRA** | **LYM%** | **MID%** | **GRA%** | **Sex** | **Age** | **Weight** | **Height** | **BMI** |
| --- | --- | --- | --- | --- | --- | --- | --- | --- | --- | --- | --- | --- | --- | --- | --- | --- | --- | --- | --- | --- | --- | --- | --- | --- | --- |
| **Blood**  **T-Hg** | 1.000 |  |  |  |  |  |  |  |  |  |  |  |  |  |  |  |  |  |  |  |  |  |  |  |  |
| **Hair**  **T-Hg** | 0.325 | 1.000 |  |  |  |  |  |  |  |  |  |  |  |  |  |  |  |  |  |  |  |  |  |  |  |
| **WBC** | 0.186 | 0.120 | 1.000 |  |  |  |  |  |  |  |  |  |  |  |  |  |  |  |  |  |  |  |  |  |  |
| **RCB** | 0.037 | -0.055 | 0.108 | 1.000 |  |  |  |  |  |  |  |  |  |  |  |  |  |  |  |  |  |  |  |  |  |
| **HGB** | 0.131 | 0.005 | 0.203 | 0.658 | 1.000 |  |  |  |  |  |  |  |  |  |  |  |  |  |  |  |  |  |  |  |  |
| **HTC** | 0.165 | 0.053 | 0.184 | 0.762 | 0.918 | 1.000 |  |  |  |  |  |  |  |  |  |  |  |  |  |  |  |  |  |  |  |
| **MCV** | 0.199 | 0.153 | 0.047 | -0.452 | 0.237 | 0.160 | 1.000 |  |  |  |  |  |  |  |  |  |  |  |  |  |  |  |  |  |  |
| **MCH** | 0.085 | 0.060 | 0.078 | -0.469 | 0.287 | 0.076 | 0.899 | 1.000 |  |  |  |  |  |  |  |  |  |  |  |  |  |  |  |  |  |
| **MCHC** | -0.208 | -0.184 | 0.096 | -0.196 | 0.200 | -0.156 | 0.126 | 0.513 | 1.000 |  |  |  |  |  |  |  |  |  |  |  |  |  |  |  |  |
| **PLT** | 0.027 | 0.280 | 0.153 | -0.107 | -0.214 | -0.168 | -0.098 | -0.151 | -0.159 | 1.000 |  |  |  |  |  |  |  |  |  |  |  |  |  |  |  |
| **PCT** | 0.057 | 0.365 | 0.218 | -0.141 | -0.239 | -0.166 | -0.052 | -0.110 | -0.196 | 0.839 | 1.000 |  |  |  |  |  |  |  |  |  |  |  |  |  |  |
| **MPV** | -0.027 | 0.014 | -0.010 | -0.009 | 0.078 | 0.079 | 0.114 | 0.145 | 0.061 | -0.469 | 0.004 | 1.000 |  |  |  |  |  |  |  |  |  |  |  |  |  |
| **PDW** | -0.068 | 0.034 | 0.000 | -0.037 | 0.046 | 0.050 | 0.098 | 0.125 | 0.036 | -0.410 | 0.045 | 0.951 | 1.000 |  |  |  |  |  |  |  |  |  |  |  |  |
| **RDW** | -0.140 | -0.156 | -0.042 | 0.357 | -0.051 | 0.038 | -0.561 | -0.537 | -0.137 | 0.095 | 0.049 | -0.134 | -0.101 | 1.000 |  |  |  |  |  |  |  |  |  |  |  |
| **LYM** | 0.019 | 0.037 | 0.488 | 0.135 | 0.238 | 0.191 | 0.049 | 0.083 | 0.116 | -0.042 | -0.014 | -0.013 | -0.007 | -0.125 | 1.000 |  |  |  |  |  |  |  |  |  |  |
| **MID** | 0.051 | 0.007 | 0.254 | -0.110 | -0.034 | -0.065 | 0.087 | 0.125 | 0.156 | -0.053 | 0.045 | 0.095 | 0.042 | 0.035 | 0.217 | 1.000 |  |  |  |  |  |  |  |  |  |
| **GRA** | 0.185 | 0.139 | 0.847 | 0.083 | 0.118 | 0.132 | 0.027 | 0.010 | -0.020 | 0.221 | 0.268 | -0.029 | 0.003 | 0.028 | 0.099 | -0.051 | 1.000 |  |  |  |  |  |  |  |  |
| **LYM%** | -0.127 | -0.060 | -0.314 | 0.044 | 0.048 | 0.028 | 0.004 | 0.014 | 0.036 | -0.199 | -0.229 | -0.009 | -0.029 | -0.082 | 0.592 | -0.016 | -0.646 | 1.000 |  |  |  |  |  |  |  |
| **MID%** | 0.012 | -0.035 | -0.081 | -0.169 | -0.097 | -0.127 | 0.102 | 0.141 | 0.153 | -0.129 | -0.056 | 0.089 | 0.027 | 0.010 | 0.044 | 0.928 | -0.362 | 0.110 | 1.000 |  |  |  |  |  |  |
| **GRA%** | 0.080 | 0.122 | 0.315 | 0.038 | 0.002 | 0.046 | -0.019 | -0.074 | -0.131 | 0.243 | 0.242 | -0.017 | 0.032 | 0.067 | -0.475 | -0.381 | 0.736 | -0.867 | -0.526 | 1.000 |  |  |  |  |  |
| **Sex** | -0.061 | -0.101 | -0.082 | 0.071 | -0.103 | -0.046 | -0.243 | -0.220 | -0.101 | -0.069 | -0.076 | 0.041 | 0.091 | 0.131 | -0.084 | -0.130 | 0.005 | -0.035 | -0.115 | 0.039 | 1.000 |  |  |  |  |
| **Age** | 0.137 | 0.032 | -0.188 | 0.007 | 0.198 | 0.208 | 0.318 | 0.214 | -0.098 | -0.179 | -0.189 | 0.012 | 0.007 | 0.086 | -0.077 | 0.018 | -0.128 | 0.031 | 0.076 | -0.034 | 0.085 | 1.000 |  |  |  |
| **Weight** | 0.088 | 0.021 | -0.104 | 0.000 | 0.232 | 0.237 | 0.364 | 0.256 | -0.056 | -0.199 | -0.191 | 0.089 | 0.075 | 0.010 | -0.036 | -0.033 | -0.025 | -0.026 | -0.015 | 0.072 | 0.012 | 0.801 | 1.000 |  |  |
| **Height** | 0.056 | -0.003 | -0.106 | -0.086 | 0.132 | 0.137 | 0.352 | 0.247 | -0.041 | -0.142 | -0.110 | 0.116 | 0.086 | 0.019 | -0.097 | 0.091 | -0.044 | -0.076 | 0.113 | 0.048 | 0.031 | 0.869 | 0.907 | 1.000 |  |
| **BMI** | 0.077 | 0.019 | -0.052 | 0.131 | 0.309 | 0.306 | 0.250 | 0.190 | -0.035 | -0.235 | -0.249 | 0.060 | 0.071 | -0.003 | 0.133 | -0.221 | -0.002 | 0.110 | -0.215 | 0.045 | -0.025 | 0.422 | 0.772 | 0.453 | 1.000 |

The Spearman correlation coefficient is shown. Values with statistical significance are highlighted in red. Blood T-Hg: µg/L; Hair T-Hg: µg/g; Age: years; Weight: kg; Height: cm, and BMI: Body Mass Index.

**XII. Supplementary Table S10.** Correlation matrix for Achi with all variables included in the study.

| **Variable** | **Blood**  **T-Hg** | **Hair**  **T-Hg** | **WBC** | **RCB** | **HGB** | **HTC** | **MCV** | **MCH** | **MCHC** | **PLT** | **PCT** | **MPV** | **PDW** | **RDW** | **LYM** | **MID** | **GRA** | **LYM%** | **MID%** | **GRA%** | **Sex** | **Age** | **Weight** | **Height** | **BMI** |
| --- | --- | --- | --- | --- | --- | --- | --- | --- | --- | --- | --- | --- | --- | --- | --- | --- | --- | --- | --- | --- | --- | --- | --- | --- | --- |
| **Blood T-Hg** | 1.000 |  |  |  |  |  |  |  |  |  |  |  |  |  |  |  |  |  |  |  |  |  |  |  |  |
| **Hair T-Hg** | 0.803 | 1.000 |  |  |  |  |  |  |  |  |  |  |  |  |  |  |  |  |  |  |  |  |  |  |  |
| **WBC** | 0.096 | 0.132 | 1.000 |  |  |  |  |  |  |  |  |  |  |  |  |  |  |  |  |  |  |  |  |  |  |
| **RCB** | 0.004 | -0.092 | -0.025 | 1.000 |  |  |  |  |  |  |  |  |  |  |  |  |  |  |  |  |  |  |  |  |  |
| **HGB** | 0.033 | 0.016 | 0.140 | 0.474 | 1.000 |  |  |  |  |  |  |  |  |  |  |  |  |  |  |  |  |  |  |  |  |
| **HTC** | 0.075 | 0.096 | 0.031 | 0.616 | 0.896 | 1.000 |  |  |  |  |  |  |  |  |  |  |  |  |  |  |  |  |  |  |  |
| **MCV** | 0.118 | 0.196 | 0.147 | -0.571 | 0.267 | 0.184 | 1.000 |  |  |  |  |  |  |  |  |  |  |  |  |  |  |  |  |  |  |
| **MCH** | 0.058 | 0.105 | 0.188 | -0.660 | 0.242 | 0.042 | 0.939 | 1.000 |  |  |  |  |  |  |  |  |  |  |  |  |  |  |  |  |  |
| **MCHC** | -0.107 | -0.169 | 0.174 | -0.427 | 0.060 | -0.269 | 0.186 | 0.474 | 1.000 |  |  |  |  |  |  |  |  |  |  |  |  |  |  |  |  |
| **PLT** | -0.129 | -0.078 | 0.083 | 0.156 | 0.008 | 0.007 | -0.186 | -0.174 | 0.010 | 1.000 |  |  |  |  |  |  |  |  |  |  |  |  |  |  |  |
| **PCT** | -0.060 | 0.006 | 0.173 | 0.168 | 0.059 | 0.095 | -0.125 | -0.127 | -0.039 | 0.868 | 1.000 |  |  |  |  |  |  |  |  |  |  |  |  |  |  |
| **MPV** | 0.161 | 0.170 | 0.139 | 0.028 | 0.121 | 0.190 | 0.125 | 0.095 | -0.065 | -0.444 | -0.007 | 1.000 |  |  |  |  |  |  |  |  |  |  |  |  |  |
| **PDW** | 0.047 | 0.061 | 0.113 | 0.013 | 0.119 | 0.162 | 0.119 | 0.105 | -0.012 | -0.386 | 0.024 | 0.912 | 1.000 |  |  |  |  |  |  |  |  |  |  |  |  |
| **RDW** | -0.085 | -0.167 | -0.155 | 0.443 | -0.150 | -0.067 | -0.652 | -0.658 | -0.236 | 0.097 | 0.049 | -0.084 | -0.079 | 1.000 |  |  |  |  |  |  |  |  |  |  |  |
| **LYM** | -0.118 | -0.058 | 0.468 | -0.090 | 0.004 | -0.089 | 0.055 | 0.093 | 0.060 | -0.023 | 0.143 | 0.310 | 0.295 | -0.210 | 1.000 |  |  |  |  |  |  |  |  |  |  |
| **MID** | -0.020 | -0.014 | 0.613 | -0.058 | 0.180 | 0.088 | 0.166 | 0.205 | 0.147 | -0.018 | 0.088 | 0.125 | 0.152 | -0.240 | 0.351 | 1.000 |  |  |  |  |  |  |  |  |  |
| **GRA** | 0.158 | 0.193 | 0.901 | 0.044 | 0.159 | 0.087 | 0.114 | 0.132 | 0.120 | 0.099 | 0.134 | 0.045 | -0.002 | -0.029 | 0.155 | 0.367 | 1.000 |  |  |  |  |  |  |  |  |
| **LYM%** | -0.195 | -0.156 | -0.539 | -0.104 | -0.157 | -0.182 | -0.084 | -0.061 | -0.075 | -0.119 | -0.082 | 0.105 | 0.112 | -0.030 | 0.427 | -0.259 | -0.741 | 1.000 |  |  |  |  |  |  |  |
| **MID%** | -0.100 | -0.107 | 0.034 | -0.066 | 0.100 | 0.063 | 0.084 | 0.108 | 0.053 | -0.123 | -0.045 | 0.097 | 0.131 | -0.189 | 0.082 | 0.783 | -0.205 | 0.083 | 1.000 |  |  |  |  |  |  |
| **GRA%** | 0.188 | 0.191 | 0.429 | 0.114 | 0.053 | 0.094 | 0.018 | -0.010 | 0.022 | 0.142 | 0.055 | -0.172 | -0.193 | 0.125 | -0.412 | -0.146 | 0.734 | -0.853 | -0.524 | 1.000 |  |  |  |  |  |
| **Sex** | -0.118 | -0.063 | 0.010 | 0.055 | -0.154 | -0.095 | -0.154 | -0.151 | -0.015 | -0.006 | 0.074 | 0.119 | 0.096 | 0.028 | 0.005 | 0.058 | -0.002 | -0.018 | 0.094 | 0.009 | 1.000 |  |  |  |  |
| **Age** | 0.028 | 0.021 | -0.082 | 0.068 | 0.067 | 0.166 | 0.039 | -0.028 | -0.173 | -0.124 | -0.013 | 0.155 | 0.181 | 0.035 | -0.273 | -0.045 | 0.032 | -0.191 | 0.040 | 0.147 | 0.068 | 1.000 |  |  |  |
| **Weight** | 0.004 | 0.054 | 0.010 | 0.091 | 0.100 | 0.225 | 0.069 | -0.028 | -0.177 | -0.158 | -0.036 | 0.270 | 0.202 | 0.050 | -0.176 | -0.009 | 0.130 | -0.221 | 0.022 | 0.176 | 0.046 | 0.777 | 1.000 |  |  |
| **Height** | 0.005 | 0.030 | 0.029 | 0.052 | 0.064 | 0.162 | 0.071 | 0.002 | -0.103 | -0.102 | 0.012 | 0.197 | 0.189 | -0.005 | -0.222 | 0.007 | 0.140 | -0.292 | 0.031 | 0.223 | 0.059 | 0.861 | 0.867 | 1.000 |  |
| **BMI** | -0.008 | 0.090 | -0.041 | 0.120 | 0.077 | 0.206 | 0.015 | -0.077 | -0.222 | -0.182 | -0.065 | 0.329 | 0.237 | 0.083 | -0.103 | -0.032 | 0.043 | -0.087 | 0.013 | 0.072 | -0.007 | 0.391 | 0.794 | 0.425 | 1.000 |

The Spearman correlation coefficient is shown. Values with statistical significance are highlighted in red. Blood Hg-T: µg/L; Hair Hg-T: µg/g; Age: years; Weight: kg; Height: cm, and BMI: Body Mass Index.


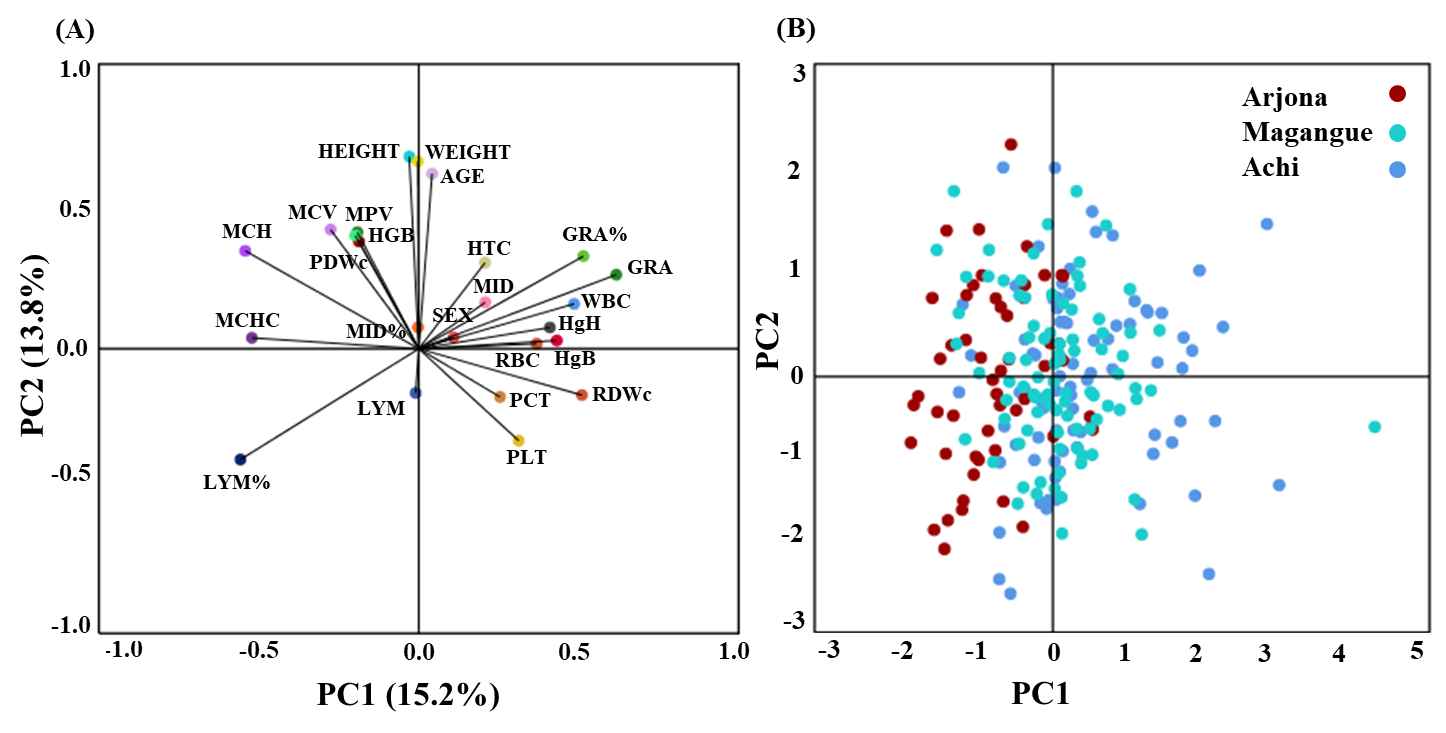


**XIII. Supplementary Figure S3.** Principal component analysis (PCA) for T-Hg concentrations in blood and hair, hematologic parameters, age, sex, and morphometric variables (A). Scatterplot of factor scores for PC1 and PC2 of analyzed variables across three sampling sites (B).
